# On the interaction between implicit statistical learning and the alternation advantage: Evidence from manual and oculomotor serial reaction time tasks

**DOI:** 10.1371/journal.pone.0318638

**Published:** 2025-02-06

**Authors:** Arianna Compostella, Marta Tagliani, Maria Vender, Denis Delfitto

**Affiliations:** 1 Department of Foreign Languages and Literatures, University of Verona, Verona, Italy; 2 Department of Cultures and Civilizations, University of Verona, Verona, Italy; University of Padova, ITALY

## Abstract

In this study, we examine how implicit statistical learning (ISL) interacts with the cognitive bias of the *alternation advantage* in serial reaction time (SRT) tasks. Our aim was to disentangle perceptual from motor aspects of learning, as well as to shed light on the cognitive sources of this alternation effect. We developed a manual (Study 1) and an oculomotor (Study 2) two-choice SRT task, with visual stimuli following the regularities of two binary artificial grammars (Fibonacci and its modification Skip). While these grammars share some deterministic transitional regularities, they differ in their probabilistic transitional regularities and distributional properties. The pattern of manual RTs in Study 1 provide evidence for ISL, showing that subjects learned the deterministic and probabilistic transitions in the two grammars. We also found a bias toward alternation (vs. repetition) in correspondence to non-deterministic points, regardless of their statistical properties in the grammars. Study 2 provides further evidence for both ISL and the alternation advantage, in terms of shorter manual RTs and higher accuracy rates of anticipatory eye movements. Saccadic responses preceding stimulus onset allow us to argue for the perceptual nature of ISL: participants detected regularities in the string by forming S-S associations based on the sequence of the perceived stimuli. Moreover, we propose that shifts in visuospatial attention preceding oculomotor programming play a role in the occurrence of the alternation advantage, and that such an effect is driven by the spatial location of the stimulus. These findings are also discussed with respect to the presence of two (possibly interacting) parsing strategies: statistical generalizations on the string vs. local hierarchical reconstruction.

## Introduction

The Serial Reaction Time task (SRT), introduced by [1], is a widely used methodology for the study of Implicit Statistical Learning (ISL), which refers to subjects’ ability to unconsciously pick up the regularities of an (artificial) grammar they are exposed to. In this paradigm, participants are exposed to sequences of stimuli, the succession of which follows specific rules. Being unaware of the presence of rules, they are asked to press specific keys as fast and accurately as they can in response to stimuli. If learning takes place, a decrease in reaction times (RTs) and/or an increase in accuracy rates are expected as the task progresses.

Despite the undisputed validity of the paradigm, which has been widely adopted to assess ISL, some issues of primary importance have not yet been fully addressed. First, the results of an SRT task cannot be taken as a pure measure of ISL, as they could be related to other external factors. Indeed, RTs might be affected by cognitive biases that are orthogonal to learning statistical regularities. For instance, it has been observed that when at least 500 ms elapse between the response and the next stimulus, participants performing a two-choice SRT task with randomized sequences of stimuli tend to respond faster to consecutive switching stimuli (e.g., ABAB), rather than consecutive repeated ones (e.g., AABB) [2, 3]. This bias, known as *alternation advantage*. Another cognitive phenomenon related to learning, expectation, and decision-making is the Perruchet effect [4, 5] which demonstrates a dissociation between conscious expectations and conditioned responses during classical conditioning. In Perruchet’s original experiment, a tone was presented alone on half the trials and followed by an air puff to the eye on the other half, eliciting anticipatory eye blinks. As the number of consecutive tone-alone trials increased, participants’ expectancy of the air puff rose, while expectancy decreased with consecutive tone-puff trials. Interestingly, conditioned responses showed the opposite pattern: participants increasingly blinked at the tone-alone trials, regardless of their conscious expectations. This suggests that automatic responses are driven by associative learning processes that operate independently of conscious cognition. Both the Perruchet effect and the alternation advantage involve expectancy in learning, influenced by a form of the *gambler’s fallacy* [6], where subjects expect alternation after repeated similar outcomes. The key distinction is that the Perruchet effect highlights a divergence between expectation (leading to a tendency towards alternation) and conditioned responses, whereas the alternation advantage effect shows how people develop expectations of alternations based on perceived patterns in randomized sequences. These phenomena illustrate the complexities of learning and expectation, revealing that human behavior often diverges from what single-process models predict [7, 8].

The alternation advantage is widely attested in the literature [9–13] but, so far, the question of how it may interact with ISL has not been directly investigated. Using the Artificial Grammar Learning (AGL) paradigm, [14] found that subjects learned an artificial language without exploiting its center-embedded structure but rather by extracting regularities from the input sequence based on alternation patterns. Participants were first exposed to sound sequences created by a phrase structure grammar that generates center-embedded structures (A^n^B^n^), instantiated in patterns such as AABB or AAABBB. After familiarization, they judged whether new auditory strings matched the previously heard patterns. Half of the new sequences followed the same grammar (A^n^B^n^), while the other half used a different grammar (AB^n^), producing patterns such as ABAB or ABABAB. Results showed that participants were not sensitive to violations in the center-embedded structures but were significantly better at detecting violations in the alternating sequences. Interestingly, participants were more sensitive to acoustic changes in longer (3-syllable) strings, contrary to expectations for center-embedded processing, where performance usually decreases with increased embedding depth. These findings suggest that participants were likely discriminating between successive alternations rather than processing the sequences as center-embedded structures. In contrast, [15] reported evidence that in 7-month-old infants learning takes place independently of possible reduplication/alternation patterns that can be detected in the input. In this study, sixteen infants were randomly assigned to either an "ABA" or "ABB" condition. Infants in the ABA condition were familiarized with sentences following an ABA pattern, while those in the ABB condition heard sentences following an ABB pattern. During testing, they were presented with consistent sentences (matching the familiarized grammar) and inconsistent sentences (matching the unfamiliar grammar). Fifteen out of 16 infants showed a preference for inconsistent sentences, as indicated by the fact that they attended longer to unfamiliar structures, regardless of reduplication or alternation patterns in the grammar. However, it is important to note that infants and adults have different cognitive and perceptual abilities, leading researchers to use distinct assessment methods for each population (e.g., verbal evaluation for adults and preferential looking patterns for infants), which makes direct comparison between these studies challenging. To the authors’ knowledge, there are no other studies that have investigated the interaction between ISL and the alternation advantage through AGL so far.

Furthermore, it is still not entirely clear what the cognitive sources of the *alternation advantage* are, as found with both manual and saccadic responses. Is it related to the alternation of the perceived target location or to subjects’ (visuo)motor responses? In a series of eye-tracking studies, [11] provided evidence for the perceptual nature of this bias, and suggested it may be related to the inhibition of return (IOR) mechanism, that is, the tendency of participants to be slower in reorienting their visual attention to a previously attended location [16]. However, since the IOR is thought to be a complex mechanism, originating from sensory and attentional components [11, 17–19], but also involving motor and oculomotor components [16, 20, 21], it is not entirely clear whether we can assert that the alternation bias is purely perceptual, or whether a motor component related to eye movements plays a specific role.

The perceptual vs. motor debate is not only about the nature of the cognitive biases that may occur in the SRT task. There is no unanimous consensus even regarding the nature of statistical learning itself. What do subjects learn? Proponents of the perceptual learning view argue that subjects build associations between stimuli, that is, based on the sequence of previous stimuli events (stimulus to stimulus, i.e., S-S) [22, 23]. On the contrary, scholars supporting a motor learning view suggest that subjects form associations between motor responses, hence, they detect regularities in the sequence of response events (response to response, i.e., R-R) [24, 25]. In addition to these two views, other theories argue that learning is neither perceptual, nor solely motor, but it involves both components, as in [26], who suggest that learning is the result of stimulus-response (S-R) type associations.

A significant limitation of previous research on ISL using the SRT task is that learning was typically assessed indirectly, relying on manual reaction times measured after stimulus onset. Since this type of task always involves an explicit motor component (i.e., key pressing), it does not allow establishing whether learning is exclusively based on associations between perceived stimuli, independently of the manual response tied to the learned component of the sequence. In the present study, we address this limitation and offer new insights into these unresolved issues by presenting the results of two two-choice SRT studies, where we directly measure potential learning effects by combining the analysis of anticipatory gaze shifts before stimulus (and thus response) onset with classical manual responses following stimulus onset.

## The current study

Our study comprised two different tasks addressing ISL of an artificial grammar in different modalities: a manual response SRT task in the visual domain (Study 1), and an eye-tracking SRT task (Study 2) in which, in addition to manual responses, we analyse anticipatory eye movements preceding stimulus presentation, aiming to measure ISL directly and thus corroborating the findings from Study 1. Oculomotor implementations of the traditional SRT task have indeed shown that saccadic eye movements reflect sequence learning [27–29]. In particular, anticipatory eye movements are considered a purer measure of ISL than manual responses, as they precede possible S-R and R-R associations [30–33]. A decrease in manual RTs is generally taken as evidence of improved anticipation (and hence learning) of the upcoming stimulus location. The analysis of participants’ predictive behaviour allows us to directly test this learning hypothesis, under the assumption that gaze shifts towards the anticipated location *prior* to the appearance of the stimulus indicate that this component of the sequence has been learned. Crucially, as anticipatory eye movements obviously precede subjects’ manual responses to the stimulus, we can also disentangle motor from perceptual aspects of learning, as well as provide relevant insights into the nature of the alternation advantage and its interaction with ISL. To date, this bias has always been discussed in relation to participants’ response behaviour (either manual or visuomotor) to stimulus presentation. Extending the analysis to anticipatory eye movements can thus shed light on possible oculomotor features of the alternation advantage that may be related to components of visuospatial attention.

In both studies, we expose subjects to sequences generated by two artificial binary grammars belonging to the so-called Lindenmayer systems: the Fibonacci grammar (Fib) and the foil Skip grammar (Skip) [34]. Previous research has investigated ISL using Fib [35–38]. Both Fib and Skip are defined by the alphabet Σ = {0, 1}. In Fib, the following rewriting rules hold: 0→1 (i.e., every 0 rewrites as 1) and 1→01 (i.e., every 1 rewrite as 01). Repeatedly applying these rules produces increasingly longer sequences of points, with each sequence representing a "generation" of the grammar. In Fib, the sequence 1 corresponds to generation 1, 01 to generation 2, 101 to generation 3, 01101 to generation 4, 10101101 to generation 5, and so on. By continuing to apply the rewriting rules, it is possible to generate potentially infinite sequences (generations) of 0s and 1s [37]. In Skip, that is obtained through a manipulation of Fib in which 0 and 1 rewrite as two non-consecutive generations of the Fib grammar, the generative rules are: 0→01 (i.e., every 0 rewrites as a 01, which corresponds to generation 2 of Fib) and 1→01101 (i.e., every 1 rewrites as 01101, which corresponds to generation 4 of Fib). Although the two grammars have a different hierarchical structure, the strings generated by Fib and Skip share the same transitional regularities:

- 0 is always followed by a 1. Hence, two 0s can never appear, *00. (We call it *First Law*: 0**1**).
- two 1s are always followed by a 0. Hence, three 1s can never appear, *111. (We call it *Second Law*: 11**0**).
- 01 can be followed by 0 or by 1. Both the trigrams 01**0** and 01**1** are possible. (We call it *Third Law*: both 01**0** and 01**1** are possible).

**Fig 1:**
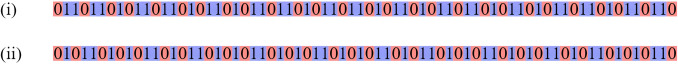
Representation of a sequence produced by the Fibonacci (i) and the Skip (ii) grammars. The 0s and 1s are highlighted in red and blue, respectively, to make the distributional difference between the two symbols in Fib and Skip more visually immediate.

Given (iii), the occurrence of 0 or 1 after the bigram 01 in Fib and Skip sequences is probabilistic. Indeed, the overall frequency of 1s and 0s in Fib and Skip strings differs. Specifically, in Fib, 1s are more frequent than 0s. In Skip, on the contrary, 0s are more frequent than 1s, as observable in Fig 1. Consequently, in Fib strings, the trigram 011 is more frequent than 010 (respectively: 62% vs. 38%), whereas in Skip strings, 010 is more frequent than 011 (respectively: 73% vs. 27%). Moreover, since Fib and Skip are binary grammars, these frequency differences are reflected in their conditional probabilities. Specifically, the likelihood of a 1 appearing after the bigram 01 is higher in Fib than in Skip. In Fib strings, *p*(1|01) = .62, while in Skip strings, *p*(1|01) = .27. Conversely, the likelihood of a 0 following 01 is higher in Skip strings than in Fib strings: *p*(0|01) = .73 in Skip, compared to *p*(0|01) = .38 in Fib. To put it shortly, in Fib sequences, 011 is more frequent than 010 and the probability that 01 is followed by a 1 is higher than the probability that 01 is followed by a 0: *p*(1|01) = .62; *p*(0|01) = .38. Conversely, in Skip sequences, 010 is more frequent than 011 and the probability that 01 is followed by a 0 is higher than the probability that 01 is followed by a 1: *p*(0|01) = .73; *p*(1|01) = .27.

Fib and Skip lend themselves optimally to our research goals: given their binary nature, they are a perfect tool for investigating the alternation advantage bias. In addition to this, their strings present regularities that could potentially be learned via conditional statistics applied to the string, allowing us to assess and shed light on the motor or perceptual nature of ISL. Finally, Fib and Skip generate strings of symbols in which the probability of having repetition between two consecutive symbols is higher than the probability of having switching, and the reverse, respectively. As we have explained, this applies both in terms of distributional frequencies and in terms of second-order transitional probabilities. As this last feature allows us to shed light on the interaction between implicit statistical learning effects and the alternation advantage bias (in the literature, we found studies suggesting that alternation effects are eliminated when probability manipulations favours repetition [2, 39]), both Fib and Skip grammars were included.

### Study 1

#### Method

*Participants*. Thirty-one native Italian speakers (7 male, 24 female) participated in Study 1. Their age ranged from 21 to 37 years (*M* = 24.76 *SD* = 6.27). Participants (both in Study 1 and in Study 2) were volunteers recruited through announcements at the University of Verona. They had normal or corrected-to-normal vision, and no history of speech, hearing, or language disorders. The two studies were approved by the local ethics committee and conducted in accordance with the standards specified in the 2013 Declaration of Helsinki. Informed consent was obtained from all participants. Participants from Study 1 received €5 as a reimburse for their participation.

**Fig 2:**
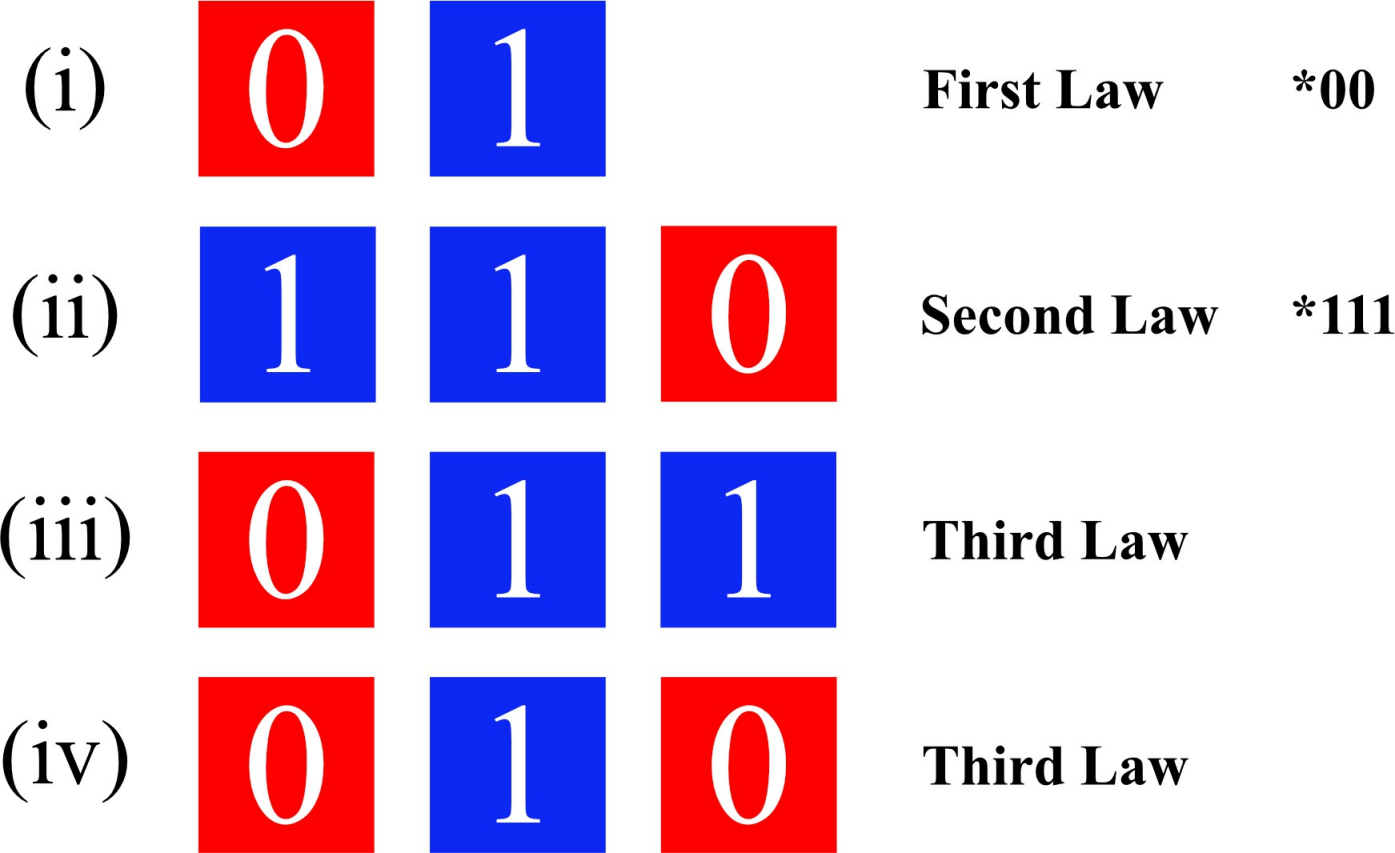
Representation of stimulus sequences reflecting the transitional regularities in Fib and Skip grammars.

*Materials*. Participants took part in a Serial Reaction Time task, which was run on a laptop using DMDX Automode version 6.3.1.4 software [40]. The stimuli consisted of a sequence of blue and red squares (dimensions 1012x536 pixels, BMP files), appearing one at a time, to the right or to the left of a computer screen. The sequence of stimuli was determined by the rules of the Fib and the foil Skip grammars: the 1s and the 0s of the grammars were respectively transmitted as blue squares and red squares (as in [36–38]; see Fig 2 below). Red squares always appeared to the computer screen’s left side, whereas blue ones to the right. The task consisted of a total of 890 trials, divided into five blocks: three initial Fib blocks (178 items each; taken from Fib generation. 14) and two final Skip blocks (97 items each, taken from Skip generation 5).

*Procedure*. Participants were tested individually, in a dimly lit and soundproof testing room at the Language, Text, and Cognition (LaTec) Laboratory of the University of Verona, sitting comfortably in front of the laptop. The experiment lasted about 30 minutes. Participants were instructed that they would have seen red and blue squares and they were asked to respond as fast as possible to them, by pressing two keys on the computer keyboard: [z] key and [m] key, in response to the red square and blue square, respectively. Participants were unaware that the stimuli followed the rules of an artificial grammar, as in [35, 38]. The fact that the sequence of stimuli was not random was told to them only at the end of the task, at the time they were asked whether they had noticed any regularities.

Each trial started with a fixation cross appearing in the middle of the screen and remaining visible for 500 ms. 250 ms elapsed between the disappearance of the fixation cross and the appearance of the blue or red square. To avoid possible confounds due to the Simon effect [41], red squares always appeared to the left side of the screen, whereas blue ones to the right side. Each square remained visible for 1000 ms, regardless of subjects’ response time. If subjects did not provide a response within that time window, the stimulus would disappear, and a new fixation cross would appear in the middle of the screen. Timing started with the onset of the square and ended with subject’s response by pressing the key.

The first part of the task consisted of a familiarization phase, intended to familiarize participants with the task. They were presented with 8 trials that did not follow the rules of the Fib, nor the Skip grammar, and received feedback on the screen based on their responses (correct/wrong). Right after this phase, participants had the opportunity to ask questions. If they did not have any questions, the testing phase began, and they did not receive feedback anymore.

*Data analysis*. To assess the presence of learning, we measured accuracy and reaction times across the task, in correspondence to the following stimuli:

- The blue square following a red square (red-**blue**, corresponding to 0**1** in Fib and Skip strings, i.e., the First Law)
- The red square following two consecutive blue squares (blue-blue-**red**, corresponding to 11**0** in Fib and Skip strings, i.e., the Second Law)
- The blue square following a red and a blue square (red-blue-**blue**, corresponding to 01**1** in Fib and Skip strings, i.e., the Third Law)
- The red square following a red and a blue square (red-blue-**red**, corresponding to 01**0** in Fib and Skip strings, i.e., the Third Law)

These regularities attested in the string have been translated into coloured squares as exemplified in Fig 2.

If learning takes place, a decrease in reaction times (and possibly an increase in accuracy) is normally observed. The research questions and predictions are the same as those of [36, 37]: since the First Law and the Second Law hold both in Fib and in Skip, if participants learn them, we expect increasingly better performances along the five blocks in terms of RTs in correspondence to the deterministic transitions (i) and (ii). Specifically, we expect that (i) will result in lower reaction times and be acquired earlier compared to (ii), as the memory load required for the former is lower than for the latter. In the first case, participants only need to remember one item (one occurrence of the red square) to predict the next, whereas in the second case, they need to track two items (two occurrences of the blue square). This expectation is consistent with the established literature [42–44] which shows that second-order transitional probabilities (i.e., the likelihood of a red square following a two-blue square sequence) are harder to learn than first-order transitional probabilities (i.e., the likelihood of a blue square following a red square).As far as (iii) and (iv) are concerned (Third Law), these stimuli do not correspond to deterministic transitions: they cannot be deterministically predicted by keeping track of the immediately preceding stimulus, not even the two immediately preceding ones. Indeed, after the sequence red-blue, there could be a blue, or a red (red-blue-**blue** and red-blue-**red** are both possible sequences on the string, corresponding to the trigrams 01**1** and 01**0** in the grammars, respectively). However, by exploiting distributional and/or conditional statistics information (see Section 2), the parser could learn to expect a 1 rather than a 0 in Fib, whereas the opposite might be true for Skip sequences. Indeed, as explained above, in Fib sequences, the trigram 011 is both more frequent and more likely, in terms of second-order transitional probabilities, than 010. Conversely, in Skip sequences, 010 is both more frequent and more likely, in terms of second-order transitional probabilities, than 011.

The same result is expected even if the parser uses two different parsing strategies that involve creating an abstract hierarchical structure from the sequence of symbols. The first strategy involves a recursive approach, where the parser forms progressively larger embedded chunks from the sequence, extending the number of deterministic transitions among progressively larger chunks [35, 45]. The second strategy entails the parser engaging in some sort of hypothetical structural reasoning [38] by applying a condition of strictly local hierarchical reconstruction by means of which if 0 precedes 1, then 0 must be contained in 1. In this way, it is shown that the parser, by locally accessing the two previous generations, is led to expect a 1, and not a 0, after the deterministic sequence 0**1**. In Fib, this structure-based prediction is in line with the probabilistic transitions on the string (01**1** is more frequent than 01**0**), whereas in Skip the prediction is disproved (since 01**0** is in fact more frequent than 01**1**).

In line with these observations, in Fib we expect to find a significant decrease in RTs for 01**1**, with RTs becoming progressively shorter across the Fib blocks, while no decrease is expected for 01**0**. On the contrary, in Skip, we do not predict improvements in RTs for 01**1**, we predict instead a decrease in RTs for 01**0**. This is because in Fib 01**1** occurs more frequently and is more likely in terms of second-order transitional probabilities than 01**0**, whereas the opposite is true for Skip. As for accuracy, since the task is fairly simple, ceiling results are expected, also based on previous studies [35–38] (where accuracy was very high despite the presence of incongruent trials). Regarding the potential effect of the alternation advantage, we aim to test (i) whether this effect occurs in our task, and (ii) how it interacts with implicit statistical learning (ISL). As we mentioned in Section 1, the interaction between the alternation advantage and ISL has not yet been directly investigated. If the alternation advantage occurs, we expect to find that 010 will show a general processing advantage over 011 in terms of shorter RTs. This should be particularly evident at the beginning of the task (Block 1), where the effect of learning statistical regularities would still be in its early stages, or not yet fully established, and therefore less pronounced. Importantly, we anticipate different RTs trends for 010 and 011 in Fib and Skip. In Fib, we expect 011 to be processed progressively faster across blocks, as it is more frequent and probable in terms of second-order transitional regularities compared to 010. Conversely, we do not expect RTs for 010 to decrease further throughout the blocks. In Fib, both distributional and conditional statistics favor repetition over alternation. Therefore, we expect implicit statistical learning in Fib to gradually weaken the alternation advantage effect. As a result, the alternation advantage should become less pronounced in Blocks 2 and 3 compared to Block 1. For Skip (Blocks 4 and 5), if implicit statistical learning occurs, we anticipate the opposite pattern compared to Fib. Here, we expect a significant decrease in RTs for 010 over the blocks. In Skip, statistical learning favors alternation over repetition, which should reinforce the alternation advantage. Consequently, we expect RTs for 010 to progressively decrease across Skip blocks (Blocks 4 and 5). Furthermore, we expect the difference in RTs between 010 and 011 to become more pronounced over time (Block 5). Specifically, RTs for 011 should no longer decrease, but instead increase, as neither the alternation advantage nor statistical properties favor the rapid processing of 011. To put it shortly, if both implicit statistical learning and the alternation advantage are present, we expect to find shorter RTs for 010 compared to 011 in both Fib and Skip. However, this difference should be less pronounced in Blocks 2 and 3 (Fib), as RTs for 011 are expected to decrease incrementally due to statistical learning effects, while we do not expect the same trend for 010. In contrast, for Skip, we predict that the RTs for 010 will not only be shorter than those for 011, but that the difference between the two will become increasingly pronounced across the blocks. In this case, the effects of statistical learning and the alternation advantage would combine, leading to a more significant difference between 010 and 011 as we progress through the Skip blocks, where both distributional and conditional statistics favor alternation.

In traditional implicit learning studies using the Serial Reaction Time Task, a control block with random stimuli is typically included to ensure the presence of learning. By comparing reaction times between blocks containing grammatical sequences and those with random sequences, we can discern the nature of any observed decrease in reaction times, isolating learning from other factors such as task habituation. While the Fibonacci grammar lacks random sequences, the presence of deterministic (i.e., 0**1** and 11**0**) and non-deterministic points (i.e., 01**1** and 01**0**) within all blocks allows us to determine whether reductions in reaction times are attributable to learning factors, excluding possible confounding factors. Hence, we conducted a preliminary analysis comparing reaction times on deterministic versus non-deterministic points for both studies. Specifically, the first analysis compared deterministic ’1s’ with non-deterministic ’1s’ within the sequence, examining reaction times for 0**1** versus 01**1**. The second analysis focused on the two types of ’0s’, comparing reaction times for 11**0 (**deterministic) and 01**0 (**non-deterministic). For both studies, the analyses showed that reaction times were significantly shorter for points in deterministic sequences (0**1** and 11**0)** than for the corresponding (01**1** and 01**0**) ones in non-deterministic sequences. We report here only the values deemed most significant. The entire analysis is available in **S1 Script**. Study 1: in 01 vs. 011 analysis we found a main effect of *point* (χ2 = 192.95, df = 1, *p* < .001); in 110 vs. 010 analysis we found a significant block*point interaction (χ2 = 23.89, df = 4, *p* < .001). Study 2: in 01 vs. 011 analysis, we found a main effect of *point* (χ2 = 87.66, df = 1, *p* < .001); in 110 vs. 010 analysis, we found a significant block*point interaction (χ2 = 13.74, df = 5, *p* = .017).

#### Results

Data were analyzed with a series of linear mixed effects regression models using lme4 and lmerTest [46, 47] in R (R Core Team 2023). For the analysis of RTs, we ran a series of Linear Mixed Models (LMM) with *RTs* as dependent variable, *Block* (1–5) and *Grammar* (Fib vs. Skip) as independent variables, and *Subject* as random intercept. As expected, accuracy rates for manual responses turned out to be at ceiling and are reported for completeness in the tables along with RTs but were not further analyzed.

**Fig 3:**
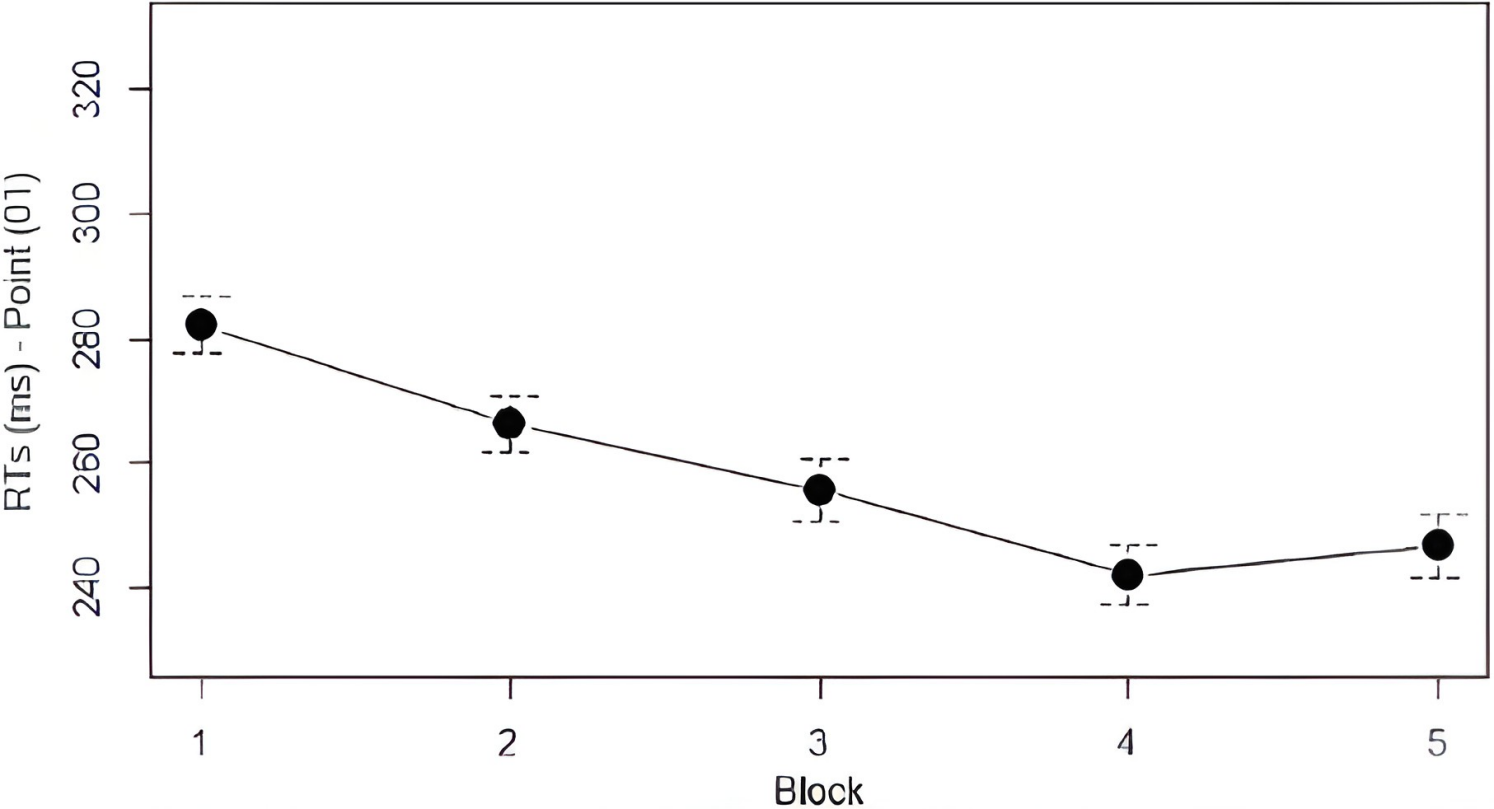
Mean RTs for point 01 by block. Error bars denote the 95% confidence interval.

*Analysis 1*: *Learning of the First Law*. To verify the learning of the First Law (0 is always followed by 1), we analyzed response times in correspondence to every instance of a blue square following a red square. A logarithmic transformation was applied to the reaction times to improve their suitability for statistical analysis [48]. Mean accuracy and log-transformed reaction times are reported in Table 1. As observable in Fig 3, RTs kept decreasing from Block 1 to Block 4, and then stabilized in Block 5. The decrease in RTs was statistically significant: we found a main effect of *Block* (χ^2^ = 326.45, df = 4, *p* < .001) with a significant decrease of RTs between Block 1 and Blocks from 2 to 5, between Block 2 and Blocks from 3 to 5, and between Block 3 and Blocks 4 and 5 (see Table 2). These results confirmed our hypothesis: participants learned the First Law, becoming faster in both Fib and Skip.

**Table 1:** Mean (SDs) log-transformed RTs and accuracy rates of each block for point 01, followed by mean (SDs) log-transformed RTs and accuracy rates in Fib and Skip.

	Block 1 (Fib)	Block 2 (Fib)	Block 3 (Fib)	Block 4 (Skip)	Block 5 (Skip)	Fib Grammar	Skip Grammar
**Log-RTs**	5.56	5.49	5.43	5.34	5.35	5.49	5.34
	(0.46)	(0.54)	(0.56)	(0.68)	(0.69)	(0.52)	(0.68)
**Accuracy**	0.99	0.98	0.97	0.96	0.95	0.98	0.95
	(0.08)	(0.14)	(0.17)	(0.20)	(0.21)	(0.13)	(0.21)

**Table 2:** Summary of significant LMM coefficients on log-transformed RTs. *M*_*diff*_ = Mean difference of raw RTs between blocks.

	*M*_*diff*_	*Β*	*SE*	*t*	*p*
Block 2—Block 1	-29.73ms	-0.08	0.02	-5.10	< .001
Block 3—Block 1	-26.57ms	-0.14	0.02	-8.73	< .001
Block 4—Block 1	-40.22ms	-0.24	0.02	-15.12	< .001
Block 5—Block 1	-35.35ms	-0.23	0.02	-14.58	< .001
Block 3—Block 2	-10.51ms	-0.06	0.02	-3.64	< .001
Block 4—Block 2	-24.17ms	-0.16	0.02	-9.90	< .001
Block 5—Block 2	-19.30ms	-0.15	0.02	-9.37	< .001
Block 4—Block 3	-13.65ms	-0.10	0.02	-6.17	< .001
Block 5—Block 3	-8.78ms	-0.09	0.02	-5.64	< .001

**Fig 4:**
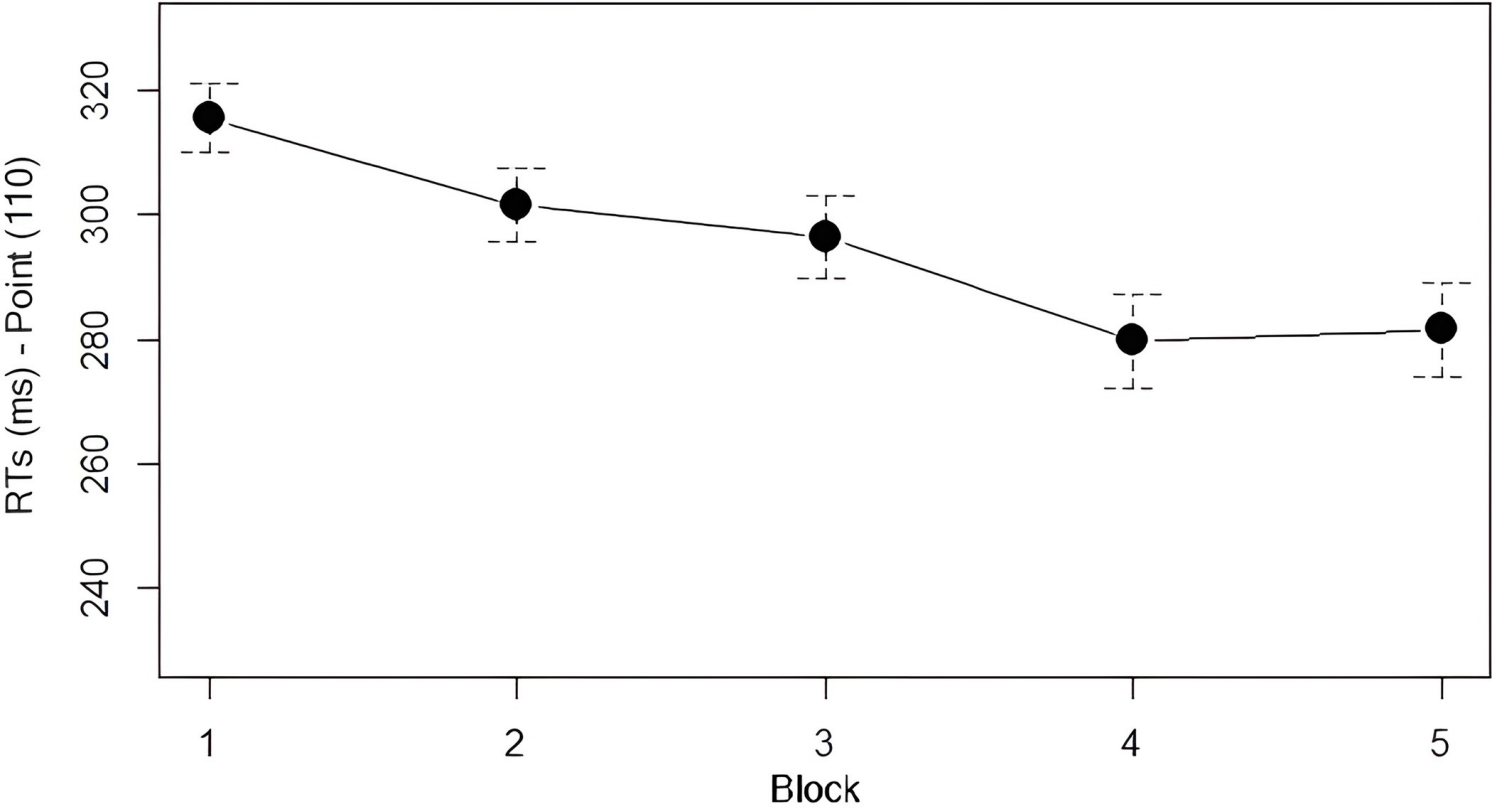
Mean RTs for point 110 by block. Error bars denote the 95% confidence interval.

*Analysis 2*: *Learning of the Second Law*. As can be seen from Fig 4, RTs at the red squares preceded by two blue squares (corresponding to the Second Law, according to which after two instances of 1 there is always a 0, *111) showed a similar trend as for the First Law: RTs progressively dropped in Blocks 2, 3, and 4, and then stabilized in the fifth and final Block (see Table 3). The decrease in RTs was statistically significant: we found a main effect of *Block* (χ^2^ = 160.52, df = 4, *p* < .001) with a significant decrease of RTs between Block 1 and Blocks from 2 to 5, between Block 2 and Blocks 4 and 5, and between Block 3 and Blocks 4 and 5 (see Table 4). As predicted, participants also learned this regularity, becoming progressively faster in both Fib and Skip.

**Table 3:** Mean (SDs) log-transformed RTs and accuracy rates of each block for point 110, followed by mean (SDs) log-transformed RTs and accuracy rates in Fib and Skip.

	Block 1 (Fib)	Block 2 (Fib)	Block 3 (Fib)	Block 4 (Skip)	Block 5 (Skip)	Fib Grammar	Skip Grammar
**Log-RTs**	5.71	5.64	5.62	5.54	5.55	5.66	5.54
	(0.31)	(0.40)	(0.39)	(0.50)	(0.49)	(0.37)	(0.50)
**Accuracy**	0.99	0.99	0.97	0.97	0.96	0.98	0.97
	(0.09)	(0.12)	(0.16)	(0.16)	(0.19)	(0.12)	(0.18)

**Table 4:** Summary of significant LMM coefficients on log-transformed RTs. *M*_*diff*_ = Mean difference of raw RTs between blocks.

	*M*_*diff*_	*β*	*SE*	*T*	*p*
Block 2—Block 1	-14.08ms	-0.07	0.01	-4.65	< .001
Block 3—Block 1	-19.12ms	-0.09	0.01	-6.05	< .001
Block 4—Block 1	-35.82ms	-0.17	0.02	-10.59	< .001
Block 5—Block 1	-33.97ms	-0.16	0.02	-10.18	< .001
Block 4—Block 2	-21.74ms	-0.10	0.02	-6.48	< .001
Block 5—Block 2	-19.89ms	-0.10	0.02	-6.05	< .001
Block 4—Block 3	-16.70ms	-0.08	0.02	-5.22	< .001
Block 5—Block 3	-14.85ms	-0.08	0.02	-4.78	< .001

**Fig 5:**
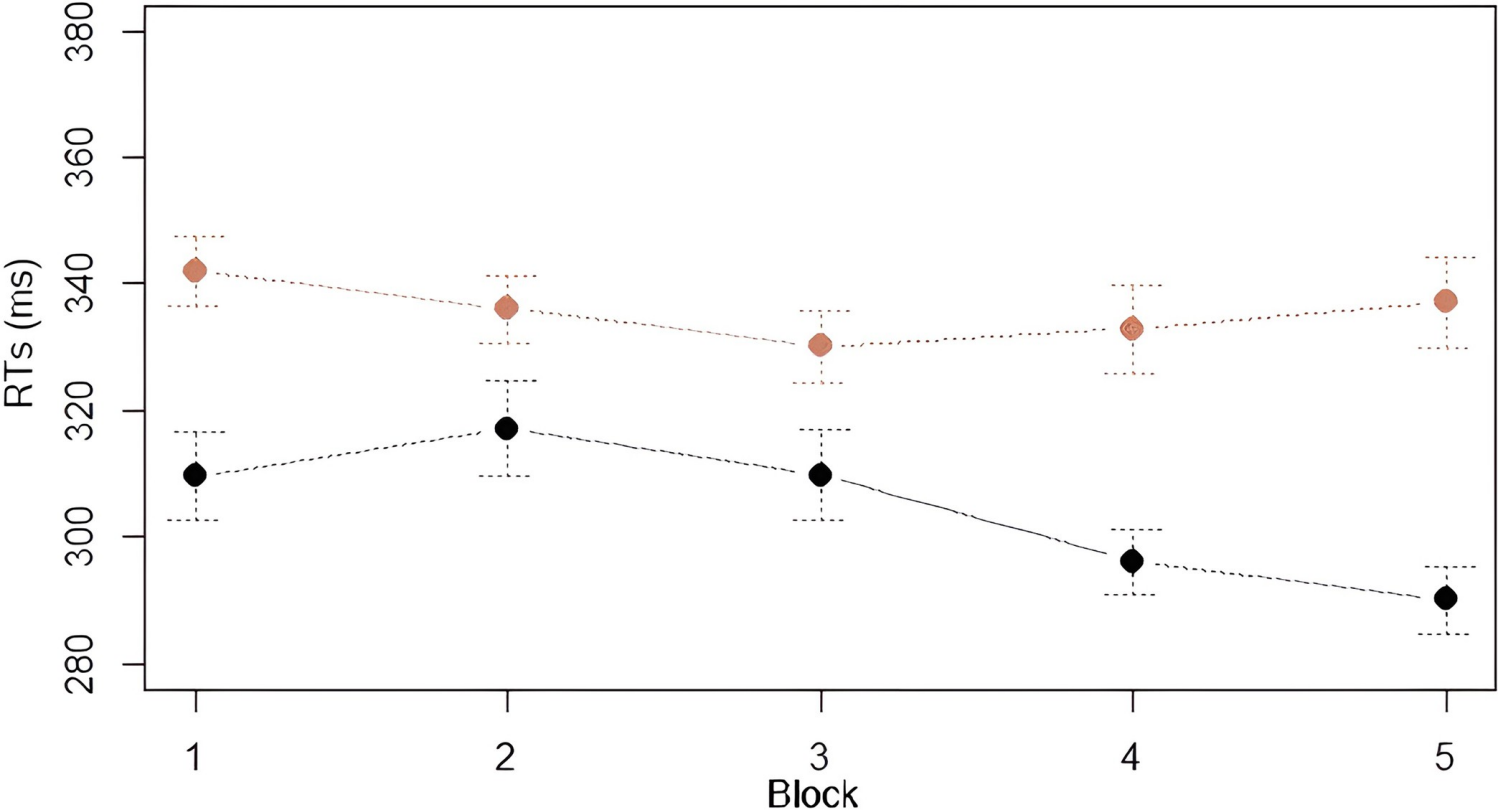
Mean RTs for points 010 (black) and 011 (red) by block. Error bars denote the 95% confidence interval.

*Analysis 3*: *Learning of the Third Law*. We analyzed and compared RTs on every instance of a blue square following a red-blue sequence (i.e., 01**1**), and every instance of a red square following a red-blue sequence (i.e., 01**0**). As seen in Fig 5, participants are always faster in responding to stimuli corresponding to the last item of the sequence 01**0** than 01**1**. Furthermore, the graph shows that RTs on 01**0** and 01**1** have different trends in Fib and Skip: RTs on 01**1** became progressively shorter along Fib blocks while increasing in Skip blocks. On the contrary, RTs on 01**0** diminished in Skip blocks, but not in Fib. Results are reported in Tables 5, 6. We assessed this statistically, by running a LMM with *RTs* as dependent variable, *Block* (1–5) and *Point* (01**0** vs. 01**1**) as independent variables with full interaction, and *Subject* as random intercept. The analysis showed a significant main effect of *Block* (χ^2^ = 89.70, df = 4, *p* < .001), with RTs becoming faster across blocks. Post-hoc comparisons with Tukey correction of p-values (emmeans()-function in R) showed a significant decrease of RTs between Block 1 and Blocks 4 and 5, between Block 2 and Blocks 4 and 5, and between Block 3 and Blocks 4 and 5 (see Table 7). We also found a main effect of *Point* (χ^2^ = 50.07, df = 1, *p* < .001), with participants being faster on 01**0** than 01**1** (301.63 ms vs. 335.87 ms, respectively). The Block*Point interaction was also significant (χ^2^ = 34.25, df = 4, *p* < .001), indicating that RTs across blocks were modulated by the type of point. Post-hoc comparisons reported a significant decrease in RTs on 01**1** from Block 1 to Block 3. RTs on 01**0** decreased significantly in the first Block of Skip (Block 4) and then continued to decrease in Block 5. A significant decrease was indeed attested between Block 1 and Blocks 4 and 5, Block 2 and Blocks 4 and 5, and between Block 3 and Blocks 4 and 5. RTs on 01**0** were significantly faster than those on 01**1** in all five blocks. Overall, these results are in line with our predictions and confirm (i) the presence of an alternation advantage: 010 was significantly faster than 011 throughout the task, both in Fib and in Skip, regardless of the statistical properties of the two grammars; and (ii) that 01**1** and 01**0** were processed following mirrored patterns between Fib and Skip: RTs for 011 decreased significantly in the Fib blocks, while those for 010 decreased significantly in the Skip blocks. This suggests that participants learned to predict the two points by exploiting the statistical properties of the Fib and Skip strings.

**Table 5:** Mean (SDs) log-transformed RTs and accuracy rates of each block for points 011 and 010, followed by mean (SDs) log-transformed RTs and accuracy rates in Fib and Skip.

	Block 1 (Fib)	Block 2 (Fib)	Block 3 (Fib)	Block 4 (Skip)	Block 5 (Skip)	Fib Grammar	Skip Grammar
**011**	5.80	5.77	5.76	5.76	5.77	5.78	5.76
**Log-RTs**	(0.29)	(0.34)	(0.29)	(0.37)	(0.35)	(0.31)	(0.36)
**011**	0.98	0.96	0.94	0.94	0.92	0.96	0.93
**Accuracy**	(0.14)	(0.20)	(0.23)	(0.24)	(0.27)	(0.20)	(0.25)
**010**	5.69	5.71	5.69	5.63	5.61	5.70	5.62
**Log-RTs**	(0.28)	(0.34)	(0.31)	(0.39)	(0.39)	(0.31)	(0.39)
**010**	0.97	0.97	0.97	0.97	0.97	0.97	0.97
**Accuracy**	(0.15)	(0.18)	(0.17)	(0.16)	(0.16)	(0.16)	(0.16)

**Table 6:** Mean (SDs) log-transformed RTs and accuracy rates for each Point*Grammar, followed by mean (SDs) log-transformed RTs and accuracy rates in Fib and Skip grammars and for points 010 and 011.

	Fib 010	Fib 011	Skip 010	Skip 011	Fib	Skip	010	011
**Log_RTs**	5.70	5.78	5.62	5.76	5.75	5.67	5.65	5.77
	(0.31)	(0.31)	(0.39)	(0.36)	(0.31)	(0.39)	(0.36)	(0.32)
**Accuracy**	0.97	0.96	0.97	0.93	0.96	0.96	0.97	0.95
	(0.16)	(0.20)	(0.16)	(0.25)	(0.19)	(0.20)	(0.16)	(0.22)

**Table 7:** Summary of significant LMM coefficients and contrasts on RTs. *M*_*diff*_ = Mean difference of raw RTs between blocks.

		*M*_*diff*_	*Β*	*SE*	*t*	*p*
**Block**	Block 4—Block 1	-21.18ms	- 0.05	0.01	-5.66	< .0001
	Block 5—Block 1	-22.97ms	- 0.06	0.01	-6.19	< .0001
	Block 4—Block 2	-19.95ms	-0.04	0.01	-4.58	< .0001
	Block 5—Block 2	-21.74ms	-0.05	0.01	-5.11	< .0001
	Block 4 –Block 3	-13.35ms	-0.03	0.01	-3.14	< .05
	Block 5 –Block 3	-15.14ms	-0.04	0.01	-3.68	< .01
Block*Point	Block 1 (01**0**–01**1**)	-32.61ms	-0.10	0.01	-7.07	< .0001
	Block 2 (01**0**–01**1**)	-18.97ms	-0.06	0.01	-4.61	< .001
	Block 3 (01**0**–01**1**)	-20.27ms	-0.07	0.01	-4.83	.0001
	Block 4 (01**0**–01**1**)	-36.81ms	-0.13	0.01	-9.44	< .0001
	Block 5 (01**0**–01**1**)	-47.05ms	-0.16	0.01	-11.72	< .0001
**011**	Block 3—Block 1	-12.06ms	-0.04	0.01	-3.30	< .05
**010**	Block 4—Block 1	-13.48ms	-0.07	0.01	-5.06	< .0001
	Block 5—Block 1	-19.49ms	-0.09	0.01	-6.53	< .0001
	Block 4—Block 2	-21.05ms	-0.08	0.01	-5.62	< .0001
	Block 5—Block 2	-27.06ms	-0.10	0.01	-7.09	< .0001
	Block 4—Block 3	-13.76ms	-0.06	0.01	-4.48	< .001
	Block 5—Block 3	-19.77ms	-0.08	0.01	-5.97	< .0001

**Fig 6:**
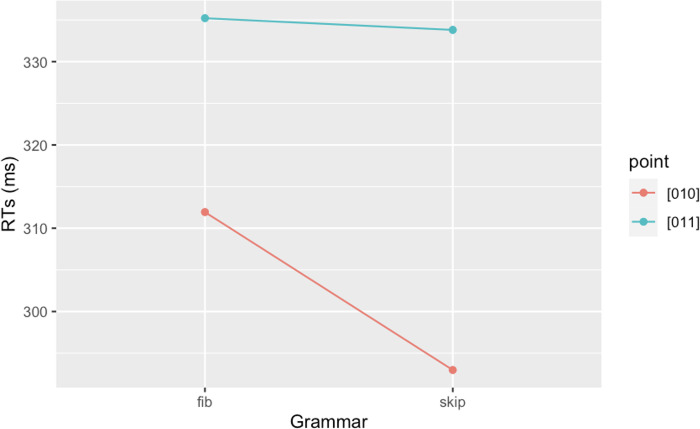
Visual representation of the Grammar*Point interaction on RTs.

To check whether there were differences in processing 01**0** and 01**1** in the two grammars Fib and Skip, we conducted a second LMM with *RTs* as dependent variable, *Grammar* (Fib vs. Skip) and *Point* (01**0** vs. 01**1**) as independent variables with full interaction, and *Subject* as random intercept. The analysis showed a significant main effect of *Point*, indicating that 01**0** and 01**1** were processed differently (χ^2^ = 91.62, df = 1, *p* < .001). Specifically, 01**0** was overall significantly faster than 01**1** (-34.23ms). We also found a significant effect of *Grammar* (χ^2^ = 85.22, df = 1, *p* < .001), with RTs in Skip being overall significantly faster than those in Fib (-19.05 ms). The Point*Grammar interaction was also significant, indicating that 01**0** and 01**1** were processed differently in Fib and Skip (χ^2^ = 27.34, df = 1, *p* < .001), as visually displayed in Fig 6. Post-hoc comparisons with Tukey correction of p-values (emmeans()-function in R) showed that RTs significantly decreased from Fib to Skip for 01**0** but not for 01**1** (*p* = .89). In Fib, RTs on 01**0** were significantly shorter than 01**1**. In Skip, RTs on 01**0** were also significantly shorter than 01**1** (see Table 8). These results, once again, align with our hypotheses.

**Table 8:** Summary of significant LMM coefficients and contrasts on log-transformed RTs. *M*_*diff*_ = mean difference of raw RTs between points or between grammars.

		*M*_*diff*_	*β*	*SE*	*t*	*p*
Point*Grammar	Fib (01**0**–01**1**)	-24.02ms	- 0.08	0.01	-9.57	< .0001
	Skip (01**0**–01**1**)	-41.88ms	-0.14	0.01	-14.94	< .0001
**010**	Skip—Fib	-19.05ms	-0.08	0.01	-9.23	< .0001

#### Discussion Study 1

Consistently with our hypotheses and confirming what has been found in previous studies [36–38], in Study 1 we found that participants acquired the First and Second Laws, by tracking conditional statistical properties on the string. This is manifested in the progressive lowering of RTs on 0**1** and 11**0**, both in Fib and Skip. RTs curves of 0**1** and 11**0** showed the same trend: for both regularities, we indeed observed a significant decrease in RTs already in the transition from Block 1 to Block 2. RTs continued to decrease significantly in all subsequent blocks (Block 3 and Block 4) and then stabilized in Block 5 (final block). We hypothesized that subjects would learn the First Law earlier than the Second Law, given the higher memory load required for the acquisition of the latter. However, in contrast with our hypothesis, we found that subjects started to become faster on 11**0** on a par with 0**1**, that is, already in Block 2. This is a clear sign that subjects became sensitive to the two regularities already in the first two blocks. However, the lower computational complexity of the First Law as compared to the Second Law is reflected in the fact that, overall, RTs on 0**1** were significantly lower than those on 11**0**. As far as 01**0** and 01**1** are concerned, RTs on 01**1** significantly dropped across Fib blocks, but not in Skip: RTs significantly decreased from Block 1 (Fib) to Block 3 (Fib). On the contrary, RTs on 01**0** significantly dropped across Skip blocks, while not in Fib (see Tables 7 and 8). We interpret this result as indicative of a statistical learning effect, since, as mentioned earlier, the overall frequency of the trigram 01**1** is higher than 01**0** in Fib, while the reverse holds for Skip. Additionally, 01**1** is more likely to occur than 01**0** in Fib in terms of second-order transitional probabilities, whereas the opposite is true for Skip. It remains to be determined whether participants relied on frequency information, transitional probability (TP) information, or both. Even small differences in TPs have been shown to impact learning, as demonstrated by [49]. Moreover, in online tasks (such as SRT tasks), TPs tend to prevail over frequency [42, 50]. Future studies could provide further insights into the nature of this result, specifically whether it can be attributed to frequency or TPs effects. Nevertheless, our results are clearly amenable to statistical learning effects, although we cannot fully clarify the precise properties of this process. Since determining the specific learning mechanisms the parser adopts in predicting non-deterministic points is a research issue that extends beyond the scope of this study (we are pursuing them in ongoing research), we will only briefly discuss it in Section 3.

Another result of primary interest we would like to focus on here, instead, is the following: although RTs on 01**0** and 01**1** showed different trends in the two grammars, RTs on 01**0** were overall significantly shorter than those on 01**1**, in every block, both in Fib and Skip. This result provides clear evidence for the presence of a cognitive bias that led subjects to be faster on 01**0**, in the absence of other possible computational strategies. In fact, subjects were found to respond faster to 01**0** than 01**1**, not only in Skip blocks, where the former trigram is both more frequent and more likely to occur in terms of transitional probabilities than the latter, but crucially also in Fib blocks, where the statistical properties of the two trigrams are reversed. This result is further supported by accuracy rates, which are overall higher for 010 than 011 (see Table 5). We are clearly facing a manifestation of the alternation advantage: subjects are facilitated in responding to consecutive switching stimuli as opposed to consecutive repeated stimuli. Summarising, in Study 1 we found that: (i) subjects became increasingly faster in responding on the occurrences of the deterministic transitions 0**1** and 11**0**. We consider this as a clear manifestation of ISL, specifically, of conditional statistics applied to the strings; (ii) subjects became increasingly faster on 01**1** in Fib blocks, while becoming increasingly faster on 01**0** in Skip blocks. This result might be attributable to different ISL mechanisms. We will briefly report on them in Section 3; (iii) subjects were overall both faster and more accurate on 01**0** than 01**1**. We interpret this result as a clear effect of the alternation advantage, especially when we consider that no other strategy (to our knowledge) could plausibly explain this result.

Two critical questions remain unanswered from Study 1: First, is the learning effect found for the First, Second, and Third Laws driven by perceptual (S-S) or motor (R-R) learning? Second, what is the nature of the alternation advantage we found on 01**0**? Is it a perceptual, or motor bias? Study 1, while revealing implicit statistical learning, relied solely on manual response times. This approach does not allow us to disentangle whether learning was based on participants’ perceptual anticipation of upcoming visual stimuli or on repeated motor responses to the sequence. In manual tasks, perceptual and motor components are indeed inextricably linked, making it difficult to determine the precise source of the attested learning effect. Study 2 is designed to overcome this limitation by assessing both anticipatory gaze shifts (to capture perceptual features of learning) and manual responses (to capture motor features). Measuring anticipatory gaze shifts allows us to directly assess potential perceptual aspects related to learning, as gaze shifts reflect participants’ ability to predict the next stimulus without requiring any motor response but solely relying on the perceived sequence of previous stimuli. By combining these two measures, we can disentangle the perceptual and motor contributions to the learning effect, providing a clearer insight into whether the observed learning and the alternation advantage are primarily perceptual or motor in nature. In this respect, Study 2 is crucial in addressing the open questions left by Study 1 and in advancing our understanding of the nature of implicit statistical learning.

### Study 2

Study 2 aims to confirm the results of Study 1 by extending the investigation to eye movements preceding stimulus presentation to discern possible confounds due to stimulus-response or response-response associations. If the findings of Study 1 do reflect ISL, we expect: i) to replicate the results as regards manual RTs; ii) to find oculomotor anticipations in correspondence of points 0**1** and 11**0**, which significantly increase as we proceed through blocks. As for the alternation advantage, we expect to replicate the advantage of the trigram 01**0** over 01**1** in terms of manual RTs, and to find similar evidence in terms of correct anticipations.

#### Method

*Participants*. Thirty-six native Italian speakers (14 male, 22 female) aged from 19;3 to 40;6 (*M* = 25;6, SD = 5;1) participated in Study 2, along the modalities discussed in Section 2.1.1 (Participants).

*Apparatus*. Participants’ eye movements were recorded at a rate of 1000 Hz using an SR Research EyeLink 1000 Plus head-mounted eye tracker connected to a 24” colour BenQ monitor for visual stimulus presentation (display resolution: 1024 x 768 pixels). The experimental procedures were implemented in Experiment Builder (SR Research Ltd., version 1.10.165). Calibration and validation procedures were carried out using a nine-point display at the beginning of the experiment and a drift correction was performed throughout the experimental session if needed. Manual responses were collected using a MilliKey Response Box.

*Stimuli*. Stimuli were blue/red squares respectively corresponding to 1 and 0, as in Study 1 (dimensions 80x80 pixels, visual angle: 2.69° x 2.69°) at the center of two rectangles (dimensions 160x160 pixels, visual angle: 5.35° x 5.35°) presented at the left/right of the central fixation point. As in Study 1, the stimuli appeared one at a time and their sequence was determined by the rules of the Fib and Skip grammars (see Section 2). Red squares always appeared to the left side of the computer screen, whereas blue ones to the right side. The task consisted of a total of 542 trials, divided into six blocks: five Fib blocks of 89 items each, for a total of 445 trials (from Fib generation 14) and one final Skip block of 97 items (from Skip generation 4). Compared with Study 1, we reduced the number of trials because the eye-tracking implementation of the task would have made it too long, thus risking that results would be affected by external factors such as boredom or mental fatigue.

*Procedure*. Manual and oculomotor responses were examined on a two-choice SRT task. Participants were told that they would see some blue or red squares appearing either in the left or in the right saccade-box. They were instructed to direct their gaze toward the left saccade-target box and press the red key (on the left side of the response box) when the stimulus was red, and toward the right saccade-target box and press the blue key (on the right side of the response box) when the stimulus was blue. To avoid the use of peripheral vision, participants were explicitly told that if they did not look at the saccade-target box, they would not be able to press the key and accomplish the task.

**Fig 7:**
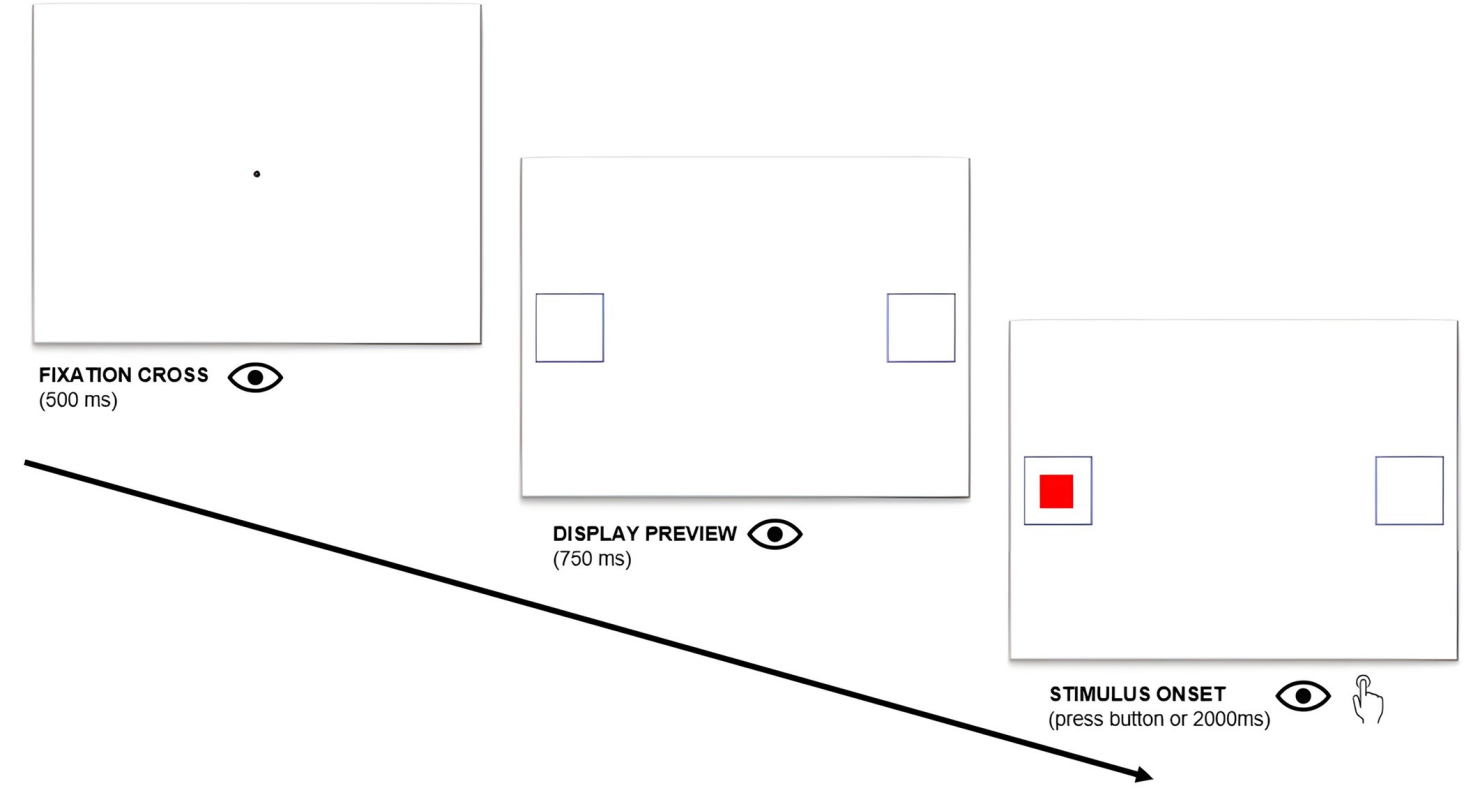
Experimental procedure of Study 2.

As shown in Fig 7, at the beginning of each trial, participants were instructed to maintain gaze on the central fixation point for at least 500ms consecutively to make the new trial start. Otherwise, a drift correction was automatically requested. Then, two empty squares, which served as saccade-target boxes, appeared on the left and on the right side of the screen and remained visible throughout the whole trial. After 750ms, the stimulus appeared at the center of either the left or the right saccade-target box and stayed in view until participants made a fixation within this area and pressed a key. Participants had 2000ms to execute the task: if they did not provide an answer within this time window, the stimulus disappeared, and the trial ended. The response-stimulus interval was thus fixed at 1250ms, of which 500ms for the initial fixation point and 750ms for the gap period between the appearance of the saccade-target boxes on the screen and that of the visual stimulus. No instructions were given for the 750ms gap period, during which participants were free to look wherever they wanted on the screen.

Participants were tested individually, in a dimly lit and soundproof testing room at the Language, Text, and Cognition (LaTec) Laboratory of the University of Verona. The testing session lasted approximately 40 minutes. There were 16 random practice trials that did not follow the rules of either grammar: in this initial phase, participants received response-contingent feedback on the screen based on their responses (correct/wrong/too slow) and had the chance to ask questions before the experiment began. They could take a short break after three experimental blocks (267 items).

*Data analysis*. Eye movement and behavioural data was extracted through DataViewer (SR Research Ltd., version 3.1.97). Analyses were conducted on two different time windows and two dependent measures were used: i) in the *preview window*, i.e., from the appearance of the saccade-target boxes to that of the stimulus, we measured the percentage of correct anticipations; ii) in the *stimulus window*, i.e., from the stimulus onset to the end of the trial, we measured manual response times.

*Correct anticipations (CA)*. This measure estimates participants’ correct anticipations of the subsequent stimulus location. Percentage of CA was assessed by tracking participants’ gaze shifts towards the correct saccade-target box during the preview window. We used the function “area of interest” in DataViewer to enlarge the two squares in order to cover the two portions of the screen to the left and right of the central fixation cross [32, 33]. For the analysis, we considered the first saccade computed after the onset of the preview window as participant’s response, whether it was towards or away from the correct saccade-target box. We automatically excluded saccades with an amplitude below 2° to discard any micromovements around the central fixation point. Trials in which no saccade was made towards either of the two saccade-target boxes (e.g., towards the top and bottom of the screen) were also discarded. With these parameters, the 46.60% of the whole eye movement dataset was included in the analysis. Note that participants were not instructed to anticipate the location of the subsequent stimulus during the preview window. Percentage of trials in which participants showed a predictive visuomotor behaviour is in line with previous works on anticipatory eye movements in sequence learning [33, 51]. If the first saccade was made towards the correct location, an anticipation score of 1 was assigned; if the location was incorrect, the anticipation score was of 0. Percentage of CA was calculated as the ratio between the number of correct anticipations over the total number of anticipations per block and type of point.

*Reaction times (RTs)*. RTs were measured in milliseconds from target stimulus onset to participants’ keypress. RTs were calculated only for those trials for which a correct anticipation was made in the preview window, representing 72.90% of the responses. This inclusion criterion was based on the well-supported hypothesis that saccadic anticipations reflect implicit learning, thereby avoiding biases related to the motor response required in the classic SRT task [27, 33]. Focusing only on trials with direct evidence of learning allowed us to achieve a more fine-grained interpretation of the learning process. Following a reviewer’s suggestion, we also analyzed trials with incorrect saccadic anticipations (**S6 Script**). While evidence of learning both the First and Second Law persisted, no significant differences were found, such as the facilitation in learning the First Law—well documented in earlier studies [36–38] and Study 1. This effect, however, emerged when we only considered trials with correct anticipations. Only latencies for correct manual answers were included in the analysis, and an additional 0.41% of trials were discarded. As said, there was a time limit for participants’ responses of 2000ms before the item disappeared: non-responses were listed as inaccurate and excluded accordingly.

#### Results

*Percentage of CA*. Data were fitted to a series of linear mixed effects regression models as in Study 1. For the CA analyses, we conducted a series of Generalized Mixed Models based on binomial distribution [48] with *Percentage of CA* as dependent variable, *Block* (1–6) as independent variable and *Subject* as random intercept.

#### Analysis 1: Learning of the First Law

**Fig 8:**
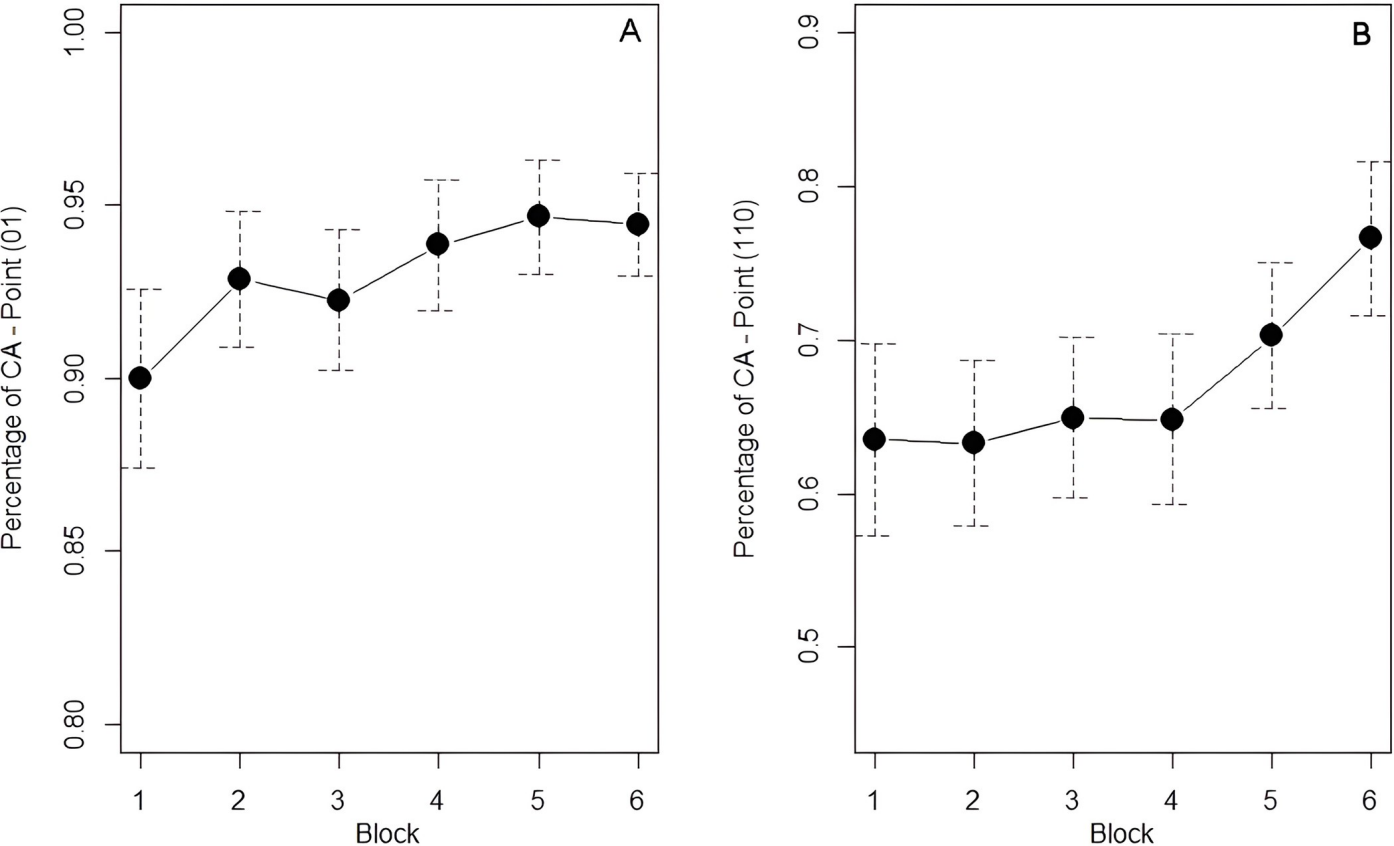
Mean percentage of correct anticipations for points 01 (A) and 110 (B) by block, with error bars denoting the 95% confidence interval.

We analyzed the correct anticipations of all 1s following a 0 across the six blocks of stimuli. As shown in Table 9, correct anticipations tend to increase across blocks. The analysis revealed a main effect of *Block* (χ^2^ = 16.21, df = 5, *p* < .001) with a significant increase of CA between Block 1 and Blocks from 4 to 6. A significant increase was also found between Block 3 and Blocks 5 and 6 (see Table 10). These results indicate that the First Law was learnt by Block 4. Moreover, as can also be observed in Fig 8A, the significance reported between Block 3 and the last two blocks suggests that participants’ anticipatory behaviour has gradually improved from the first block of the task.

**Table 9:** Mean (SDs) of correct anticipations of each block for point 01, followed by mean (SDs) of correct anticipations in Fib and Skip grammars.

Block 1 (Fib)	Block 2 (Fib)	Block 3 (Fib)	Block 4 (Fib)	Block 5 (Fib)	Block 6 (Skip)	Fib Grammar	Skip Grammar
0.90	0.93	0.92	0.94	0.95	0.94	0.93	0.94
(0.30)	(0.26)	(0.27)	(0.24)	(0.22)	(0.23)	(0.26)	(0.23)

**Table 10:** Summary of significant GLMM coefficients on correct anticipations.

	*M*_*diff*_	*β*	*SE*	*z*	*p*
Block 4—Block 1	+4%	.53	.23	2.28	.02
Block 5—Block 1	+5%	.73	.23	3.16	< .001
Block 6—Block 1	+4%	.71	.22	3.28	< .001
Block 5—Block 3	+3%	.48	.23	2.10	.03
Block 6—Block 3	+2%	.46	.21	2.15	.03

#### Analysis 2: Learning of the Second Law

We analyzed the correct anticipations of all 0s following a 1–1 sequence across the six blocks of stimuli. As shown in Table 11, correct anticipations increase considerably in the last two blocks. The analysis revealed a main effect of *Block* (χ^2^ = 22.25, df = 5, *p* < .001), indicating that the percentage of correct anticipations in Block 6 was significantly higher than in the other five blocks. A nearly significant difference was also attested between Block 4 and Block 5 (see Table 12). These results indicate that the Second Law was learnt between Block 5 and Block 6. A steeper increase of correct anticipations can indeed be observed for the last two blocks in Fig 8B.

**Table 11:** Mean (SDs) of correct anticipations of each block for point 110, followed by mean (SDs) of correct anticipations in Fib and Skip grammars.

Block 1 (Fib)	Block 2 (Fib)	Block 3 (Fib)	Block 4 (Fib)	Block 5 (Fib)	Block 6 (Skip)	Fib Grammar	Skip Grammar
0.64	0.63	0.65	0.65	0.70	0.77	0.66	0.77
(0.48)	(0.48)	(0.48)	(0.48)	(0.46)	(0.42)	(0.48)	(0.42)

**Table 12:** Summary of significant GLMM coefficients on correct anticipations.

	*M*_*diff*_	*β*	*SE*	*z*	*p*
Block 6—Block 1	+13%	.88	.24	3.76	< .001
Block 6—Block 2	+14%	.83	.22	3.80	< .001
Block 6—Block 3	+12%	.69	.22	3.16	< .01
Block 6—Block 4	+12%	.89	.22	3.99	< .001
Block 6—Block 5	+7%	.51	.22	2.34	.02
Block 5—Block 4	+5%	.38	.20	1.92	.054

#### Analysis 3: Learning of the Third Law

**Fig 9:**
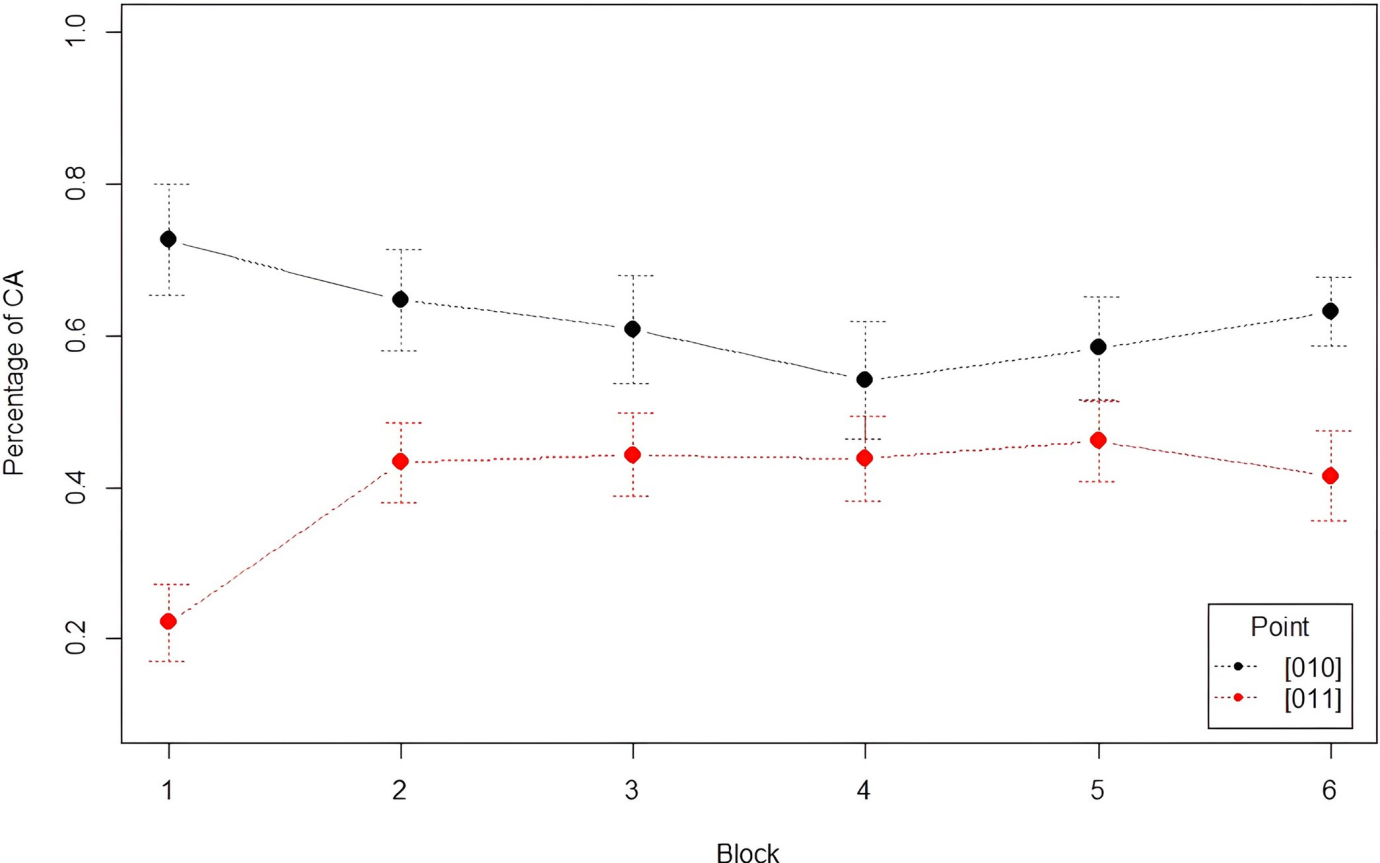
Mean percentage of correct anticipations for points 011 and 010 by block, with error bars denoting the 95% confidence interval.

We analyzed the correct anticipations across blocks and grammars comparing point 01**1**, i.e., each blue square following a red-blue sequence, with point 01**0**. Means of correct anticipations across blocks and grammars are reported in Table 13. As shown in Fig 9, correct anticipations for point 01**0** are always higher than for point 01**1**. In addition, the two points exhibit a specular behaviour: for point 01**0**, correct anticipations are very high in Block 1 and tend to decrease until Block 4 only to increase again towards the final blocks; for point 01**1**, they are, instead, very low in Block 1: they rise in Block 2, from where they stabilize until a new decrease in Block 6.

**Table 13:** Mean (SDs) of correct anticipations of each block for points 010 and 011, followed by mean (SDs) of correct anticipations in Fib and Skip grammars.

Point	Block 1 (Fib)	Block 2 (Fib)	Block 3 (Fib)	Block 4 (Fib)	Block 5 (Fib)	Block 6 (Skip)	Fib Grammar	Skip Grammar
**010**	0.73	0.65	0.61	0.54	0.58	0.63	0.62	0.63
	(0.45)	(0.48)	(0.49)	(0.50)	(0.49)	(0.48)	(0.49)	(0.48)
**011**	0.22	0.43	0.44	0.44	0.46	0.41	0.41	0.41
	(0.42)	(0.50)	(0.50)	(0.50)	(0.50)	(0.49)	(0.49)	(0.49)

A GLMM was conducted with *Percentage of CA* as dependent variable, *Block* (1–6) and *Point* (01**0** vs. 01**1**) as independent variables with full interaction, and *Subject* as random intercept. The analysis showed a significant main effect of *Block* (χ^2^ = 13.48, df = 5, *p* = .02), with a significant increase of correct anticipations between Block 1 Blocks from 3 to 6. Correct anticipations decreased significantly between Block 2 and Block 4, while they increased between Block 4 and Block 6 (see Table 14). There was also a main effect of *Point* (χ^2^ = 87.39, df = 1, *p* < .001), with participants making more correct anticipations with point 01**0** than with point 01**1** (62% vs. 41%, respectively) in all blocks except Block 4 and Block 5. The Block*Point interaction was also significant (χ^2^ = 43.30, df = 5, *p* < .001), indicating that the percentage of CA across blocks was modulated by the type of point, in that they increased for point 01**1** but decreased for point 01**0**. Post-hoc comparisons with Tukey correction of p-values (emmeans()-function in R) showed an important increase of CA for point 01**1** between Block 1 and all other blocks. In contrast, the drop in CA attested for point 01**0** reached significance only between Block 1 and Block 4. The transition from Block 5 (Fib) to Block 6 (Skip) was not significant for either point.

**Table 14:** Summary of significant GLMM coefficients and contrasts on correct anticipations.

		*M*_*diff*_	*β*	*SE*	*z*	*p*
**Block**	Block 3—Block 1	+10%	.54	.24	2.27	.02
	Block 4—Block 1	+7%	.83	.24	3.36	< .001
	Block 5—Block 1	+11%	.65	.23	2.77	< .01
	Block 6—Block 1	+15%	.45	.21	2.13	.03
	Block 4—Block 2	+4%	.51	.22	2.07	.04
	Block 6—Block 4	+8%	.38	.19	2.02	.04
Block*Point	Block 1 (01**0**–01**1**)	+51%	2.23	.24	9.35	< .001
	Block 2 (01**0**–01**1**)	+22%	.88	.19	4.76	< .001
	Block 3 (01**0**–01**1**)	+17%	.68	.19	3.57	.02
	Block 6 (01**0**–01**1**)	+22%	.88	.16	5.61	< .001
**011**	Block 2—Block 1	+21%	-.98	.19	-5.26	< .001
	Block 3—Block 1	+22%	-1.01	.19	-5.36	< .001
	Block 4—Block 1	+22%	-.99	.19	-5.21	< .001
	Block 5—Block 1	+24%	-1.09	.19	-5.86	< .001
	Block 6—Block 1	+19%	-.90	.19	-4.63	< .01
**010**	Block 4—Block 1	-19%	.83	.25	3.63	.03

Another GLMM was conducted with *Percentage of CA* as dependent variable, *Grammar* (Fib vs. Skip) and *Point* (01**0** vs. 01**1**) as independent variables with full interaction, and *Subject* as random intercept. Given the uneven number of blocks across the two grammars (5 Fib vs. 1 Skip), all the Fib blocks were considered together in the latter analysis. The analysis showed a significant main effect of *Point* (χ^2^ = 131.35, df = 1, *p* < .001) but not of *Grammar* (*p* = .64), with a comparable percentage of correct anticipations made in Fib and Skip. The Point*Grammar interaction was not added as it did not contribute to the model’s fit.

*Reaction times (RTs)*. We ran a series of linear mixed effects regression models with *RTs* as dependent variable, *Block* (1–6) as independent variable and *Subject* as random intercept. As in Study 1, reaction times were log-transformed. Accuracy rates for manual responses turned out to be at ceiling, as in Study 1, and are reported for completeness in the tables along with RTs but were not further analyzed.

#### Analysis 1: Learning of the First Law

**Fig 10:**
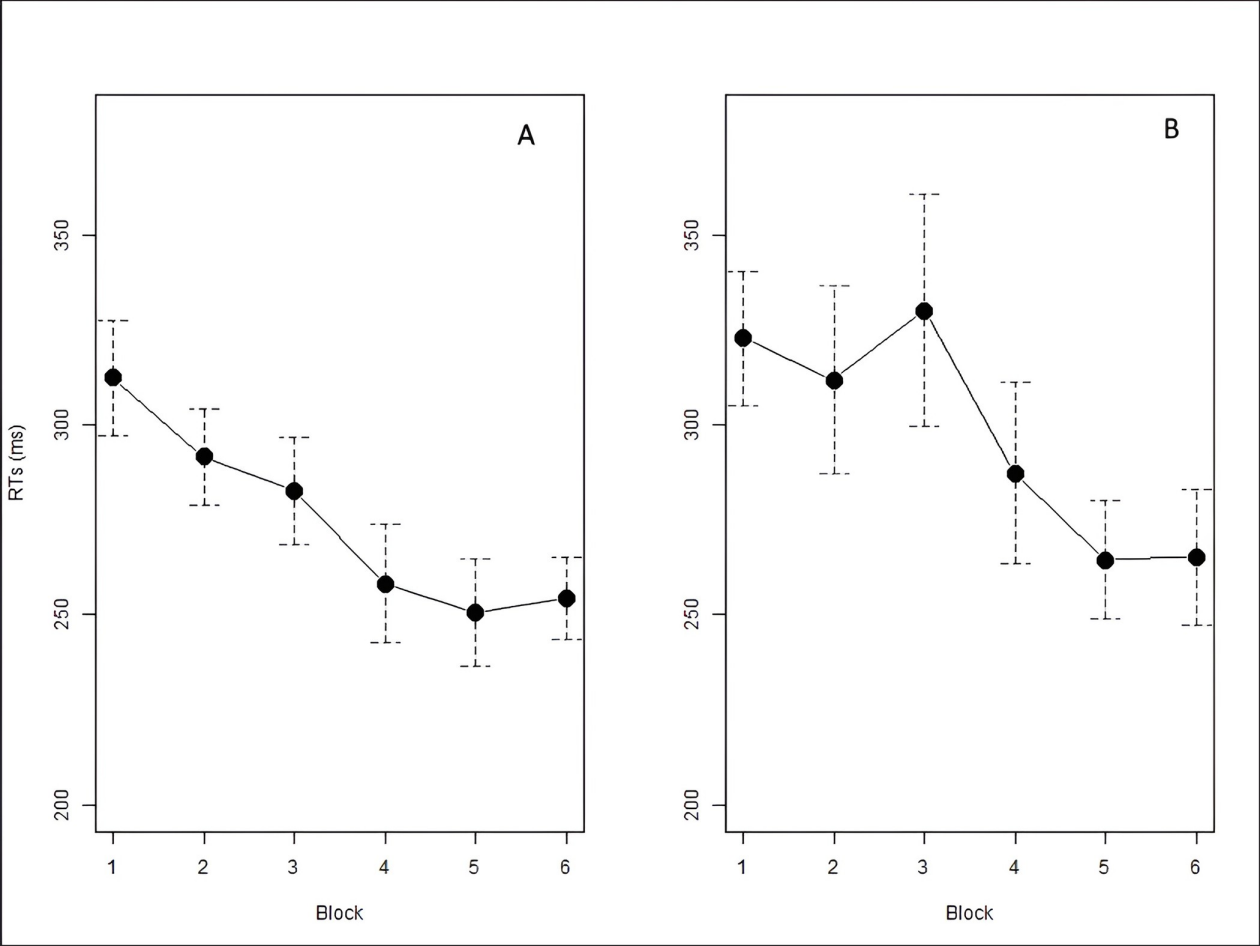
Mean RTs (ms) for points 01 (A) and 110 (B) by block, with error bars denoting the 95% confidence interval.

As shown in Table 15, RTs decrease significantly over the first five blocks, then level off when moving from Block 5 (Fib) to Block 6 (Skip). The trend is clearly observed in Fig 10A. The analysis revealed a main effect of *Block* (χ^2^ = 116.48, df = 5, *p* < .001) with a significant decrease of RTs between Block 1 and Blocks from 2 to 6. A significant decrease was also found between Block 2 and Blocks from 4 to 6, and between Block 3 and Blocks from 4 to 6 (see Table 16). These results provide further evidence of learning of the First Law, as participants become (gradually) faster in reacting to the 1s following a 0 from Block 2 onwards.

**Table 15:** Mean (SDs) log-transformed RTs and accuracy rates of each block for point 01, followed by mean (SDs) log-transformed RTs and accuracy rates in Fib and Skip grammars.

	Block 1 (Fib)	Block 2 (Fib)	Block 3 (Fib)	Block 4 (Fib)	Block 5 (Fib)	Block 6 (Skip)	Fib Grammar	Skip Grammar
**Log-RTs**	5.67	5.59	5.55	5.43	5.33	5.40	5.5	5.4
	(.35)	(.54)	(.53)	(.59)	(.85)	(.69)	(.62)	(.69)
**Accuracy**	1.00	1.00	0.99	1.00	1.00	1.00	1.00	1.00
	(0.00)	(0.00)	(0.06)	(0.00)	(0.00)	(0.00)	(0.03)	(0.00)

**Table 16:** Summary of significant LMM coefficients on log-transformed RTs. *M*_*diff*_ = mean difference of raw RTs between blocks.

	*M*_*diff*_	*β*	*SE*	*t*	*p*
Block 2—Block 1	-20.74 ms	-0.08	0.05	-2.25	.02
Block 3—Block 1	-29.73 ms	-0.11	0.04	-2.84	< .01
Block 4—Block 1	-54.18 ms	-0.22	0.04	-5.84	< .001
Block 5—Block 1	-61.79 ms	-0.33	0.04	-8.93	< .001
Block 6—Block 1	-58.18 ms	-0.25	0.03	-7.06	< .001
Block 4—Block 2	-33.44 ms	-0.14	0.03	-3.92	< .001
Block 5—Block 2	-41.05 ms	-0.24	0.03	-7.22	< .001
Block 6—Block 2	-37.44 ms	-0.16	0.03	-5.12	< .001
Block 4—Block 3	-24.45 ms	-0.11	0.04	-3.24	< .01
Block 5—Block 3	-32.06 ms	-0.22	0.03	-6.48	< .001
Block 6—Block 3	-28.45 ms	-0.14	0.03	-4.37	< .001

#### Analysis 2: Learning of the Second Law

In Fig 10B, we can observe a decrease in RTs from Block 4 onwards. Results are shown in Table 17. The analysis revealed a main effect of *Block* (χ^2^ = 33.99, df = 5, *p* < .001) indicating that RTs were significantly shorter in Block 5 and Block 6 than in the first four blocks (see Table 18). These results suggest that the Second Law was learnt, as participants become faster in reacting to the 0s following a 1–1 sequence around Block 5.

**Table 17:** Mean (SDs) log-transformed RTs and accuracy rates of each block for point 110, followed by mean (SDs) log-transformed RTs and accuracy rates in Fib and Skip grammars.

	Block 1 (Fib)	Block 2 (Fib)	Block 3 (Fib)	Block 4 (Fib)	Block 5 (Fib)	Block 6 (Skip)	Fib Grammar	Skip Grammar
**Log-RTs**	5.79	5.70	5.65	5.62	5.55	5.46	5.65	(5.46)
	(.32)	(.48)	(.72)	(.41)	(.51)	(.81)	(.52)	(.81)
**Accuracy**	1.00	1.00	1.00	0.99	0.99	0.99	1.00	0.99
	(0.00)	(0.07)	(0.07)	(0.07)	(0.09)	(0.10)	(0.07)	(0.10)

**Table 18:** Summary of significant LMM coefficients on log-transformed RTs. *M*_*diff*_ = mean difference of raw RTs between blocks.

	*M*_*diff*_	*β*	*SE*	*t*	*p*
Block 5—Block 1	-58.3 ms	-0.22	0.06	-3.60	< .001
Block 5—Block 2	-47.3 ms	-0.13	0.05	-2.32	< .01
Block 5—Block 4	-22.72 ms	-0.14	0.06	-2.40	< .01
Block 6—Block 1	-57.67 ms	-0.32	0.06	-5.04	< .001
Block 6—Block 2	-46.69 ms	-0.23	0.06	-3.90	< .001
Block 6—Block 3	-65.1 ms	-0.19	0.06	-3.30	< .001
Block 6—Block 4	-22.08 ms	-0.24	0.06	-3.97	< .001

#### Analysis 3: Learning of the Third Law

**Fig 11:**
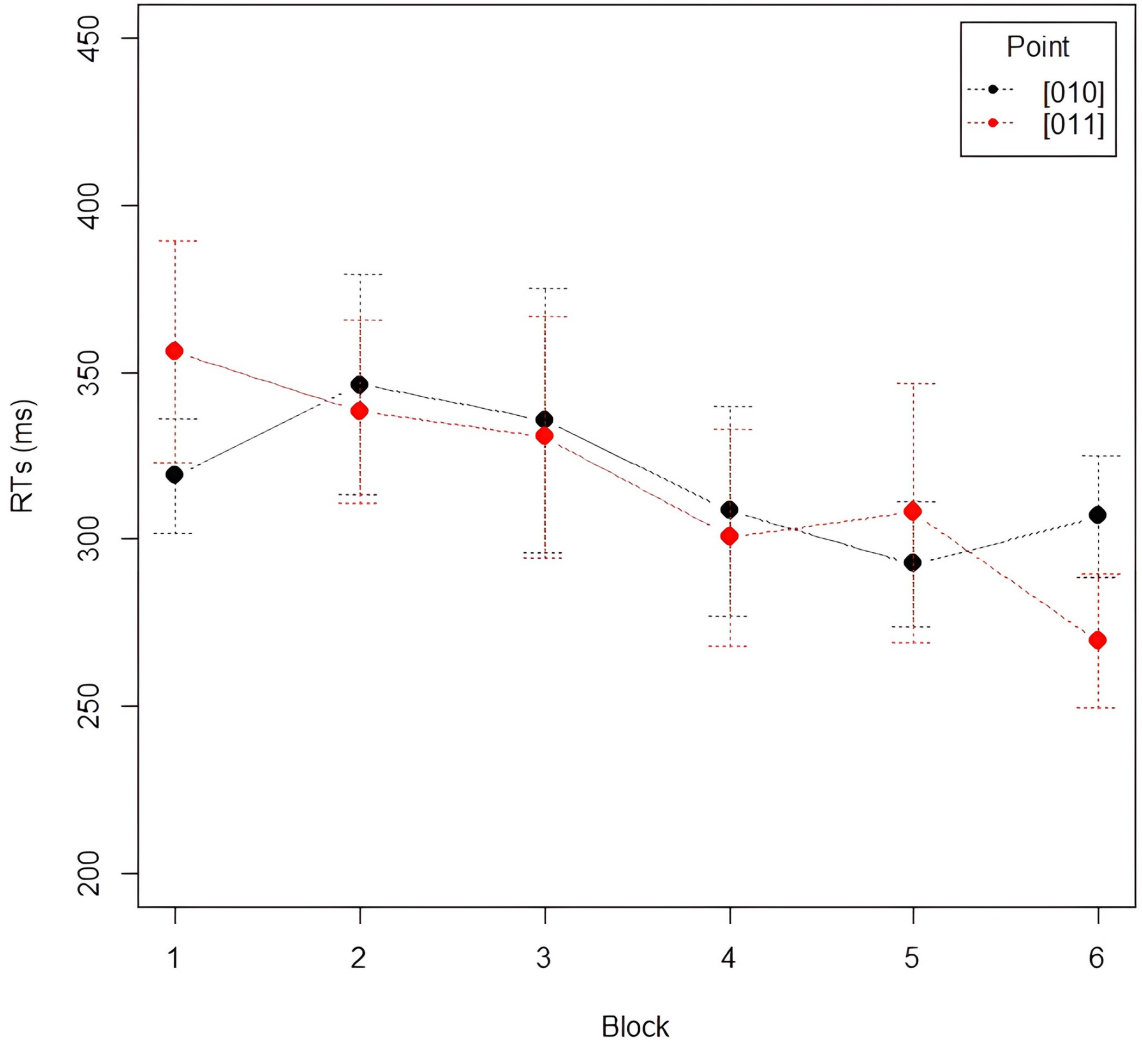
Mean RTs (ms) for points 011 and 010 by block, with error bars denoting the 95% confidence interval.

As shown in Fig 11, reaction times for the two points are very similar across blocks. However, in Block 1 we observe higher RTs for point 01**1** than for point 01**0**. In Block 2, reaction times for point 01**1** tend to decrease while those for point 010 increase: the same trend is found when moving from Block 5 to Block 6. Means of RTs across blocks and grammars are reported in Table 19.

**Table 19:** Mean (SDs) log-transformed RTs and accuracy rates of each block for points 010 and 011, followed by mean (SDs) log-transformed RTs and accuracy rates in Fib and Skip grammars.

	Block 1 (Fib)	Block 2 (Fib)	Block 3 (Fib)	Block 4 (Fib)	Block 5 (Fib)	Block 6 (Skip)	Fib Grammar	Skip Grammar
**010**	5.76	5.80	5.74	5.69	5.67	5.67	5.73	5.67
**Log-RTs**	(.29)	(.35)	(.41)	(.32)	(.36)	(.38)	(.35)	(.38)
**010**	1.00	1.00	0.99	0.99	1.00	0.99	1.00	0.99
**Accuracy**	(0.00)	(0.00)	0.09	0.10	(0.00)	0.10	(0.00)	0.10
**011**	5.86	5.79	5.75	5.74	5.71	5.71	5.76	5.71
**Log-RTs**	(.30)	(.40)	(.62)	(.40)	(.57)	(.47)	(.48)	(.47)
**011**	1.00	1.00	1.00	0.99	0.99	0.97	1.00	0.97
**Accuracy**	(0.00)	(0.00)	(0.00)	0.09	0.11	0.16	(0.00)	0.16

A LMM was conducted with *RTs* as dependent variable, *Block* (1–6) and *Point* (01**0** vs. 01**1**) as independent variables with full interaction, and *Subject* as random intercept. The analysis showed a significant main effect of *Block* (χ^2^ = 18.81, df = 5, *p* < .01), with a reduction of RTs between Block 1 and Blocks 5 and 6, Block 2 and Blocks 4, 5 and 6, and between Block 3 and Block 5 (see Table 20). There was no main effect of *Point* (*p* = .85), indicating that participants reacted similarly fast when they encountered points 01**0** and 01**1** in the sequence (318.09 ms vs. 317.16 ms, respectively). The Block*Point interaction was not added as it did not contribute to the model’s fit.

**Table 20:** Summary of significant LMM coefficients on log-transformed RTs. *M*_*diff*_ = mean difference of raw RTs between blocks.

	*M*_*diff*_	*β*	*SE*	*t*	*p*
Block 5 –Block 1	-37,36 ms	-0.11	0.04	-2.80	< .001
Block 6 –Block 1	-49,56 ms	-0.09	0.04	-2.57	< .01
Block 4 –Block 2	-37,73 ms	-0.07	0.04	-2.05	.04
Block 5 –Block 2	-41.97 ms	-0.12	0.03	-3.45	< .001
Block 6 –Block 2	-54.17ms	-0.10	0.03	-3.23	< .001
Block 5 –Block 3	-32.70 ms	-0.07	0.03	-1.99	.04

Another LMM was conducted with *RTs* as dependent variable, *Grammar* (Fib vs. Skip) and *Point* (01**0** vs. 01**1**) as independent variables with full interaction, and *Subject* as random intercept. The analysis showed no main effect of *Point* (*p* = .97). We found a significant main effect of *Grammar* (χ^2^ = 4.35, df = 1, *p* = .03), with a decrease in RTs switching from Fib (322.87 ms) to Skip (288.07 ms). Such decrease is independent of the type of point, as indicated by the lack of a significant Point*Grammar interaction.

#### Discussion Study 2

The increase in correct anticipations with points 0**1** and 11**0** provides direct evidence of sequential learning of the First and Second Law. In line with our hypotheses, the First Law seems to be acquired earlier than the Second Law. Reaction time data further support this learning hypothesis, as we observed a significant improvement in participants’ performance (either visuomotor or manual) between Blocks 2 and 3 for the First Law and between Blocks 5 and 6 for the Second Law. Note that the percentage of correct anticipations for point 0**1** stands at 90% already in Block 1, reaching 95% in Block 5. This finding suggests the presence of a bias toward alternation also in predictive visuomotor behaviour that interferes with the application of the First Law (which is easily learned), leading to the remarkably high percentage of correct anticipations. Nonetheless, if this outcome were exclusively due to an alternation bias, we would expect very high percentages also for point 11**0**: instead, the percentage of correct anticipations for this latter point is considerably lower, standing at 64% in Block 1 and remaining below 80% throughout the task. Taken together, these results point to an interplay between the alternation advantage and ISL, that is more evident with the First Law–the easiest to master as it applies to bigrams. As suggested by a reviewer, however, to precisely determine whether the high correct anticipation rates from the first block and their increase across blocks reflects not only conditional statistics but also an alternation advantage, a future study could compare the results of a deterministic first-order rule with alternation (e.g., *p*(y|z) = 1, as in the case of *p*(1|0) = 1 in the present study) with a deterministic first-order rule with repetition (e.g., *p*(x|x) = 1).

Participants made more correct anticipations for point 01**0** than for point 01**1** regardless of their statistical properties (in terms of distributional or transitional probabilities) in the underlying grammar. This finding is quite remarkable in Block 1 (Fib): despite point 01**1** being more frequent than point 01**0** in Fib, and despite 011 being more likely than 010 in terms of second-order transitional probabilities, correct anticipations for the latter outnumber those for the former by 51%. This effect cannot thus be the result of implicit statistical learning applied to the string; however, it can be explained by taking into account i) the presence of an alternation bias that makes participants more likely to expect a 0 rather than a 1 after a 01 bigram ii) an over-application of the First Law. Reaction time data showed that participants reacted at a similar pace when presented with the two types of point. Again, their frequency in the underlying grammar seems not to have affected the manual response behaviour. The fact that RTs are shorter in Skip than in Fib (regardless of the point type, as evidenced by the lack of a significant interaction) can arguably indicate that participants simply became faster in performing the task. These results do not confirm the alternation advantage of point 01**0** over point 01**1** either, as found in participants’ visuomotor behaviour during the preview window. However, the results of the two analyses give us a clearer picture of the overall task performance. In the transition from Block 1 to Block 2, we noticed that correct anticipations improved for point 01**1** and worsened significantly for point 01**0**. At first, participants arguably expected more 01**0** than 01**1** sequences, as a result of the effects described above. As the task progressed, they realized that this strategy did not allow for the correct extraction of all the string regularities. This, along with a greater exposure to Fib grammar, where 01**1** is more frequent, led participants to make more correct anticipations for point 01**1** at the expense of those for point 01**0**, although the overall advantage for point 01**0** remained. This variation is reflected in reaction times: between these two blocks, participants tended to become faster with point 01**1** and slower with point 01**0,** albeit not significantly.

## General discussion and conclusions

This work had a twofold aim: first, to shed light on the nature of ISL, disentangling perceptual from motor aspects of learning; second, to explore whether a cognitive bias known as the alternation advantage may interfere with it. We developed two two-choice SRT tasks with visual stimuli following the regularities of two artificial grammars, Fib and Skip, which share some deterministic transitional regularities (First and Second Law) but display different distributional properties and different transitional probabilities for probabilistic points (Third Law) (see Section 2). After finding positive evidence for both the presence of ISL and the alternation advantage in the manual SRT task (Study 1), we exploited the eye-tracking methodology to disentangle the nature of ISL and the alternation advantage by measuring not only manual responses upon stimulus presentation (considered an indirect measure of learning) but also saccadic eye movements preceding its onset (Study 2). Results of Study 2 confirmed and extended those of Study 1, providing further evidence that subjects learned the First and the Second Law by tracking conditional statistical properties on the string. We attribute the observed learning differences between the two transitional regularities to the different memory load required for their computation. An advantage of point 01**0** over 01**1** has been attested in terms of shorter reaction times (Study 1) and more correct anticipations (Study 2). Crucially, this result was observed in both grammars regardless the distributional frequency of the two points, and we interpreted it as a manifestation of the alternation advantage.

To our knowledge, this is the first AGL study to assess ISL and the alternation advantage through anticipatory eye movements. The recording of eye movement data from the preview window, preceding stimulus onset, is particularly powerful because it allows us to measure participants’ perceptual anticipation of upcoming stimuli independently from manual responses. This method provides a direct, real-time reflection of how participants process and anticipate stimuli through implicit statistical learning. By observing anticipatory gaze shifts, we specifically assess perceptual learning and effectively disentangle it from motor learning. Unlike manual responses, which might blend perceptual and motor components, anticipatory gaze shifts isolate perceptual processing and offer a clearer view of the nature of the learning effects. This approach enabled us to clearly determine whether the learning observed was driven by perceptual stimulus-stimulus (S-S) associations rather than motor-based response-response (R-R) or stimulus-response (S-R) associations. If the learning were based on R-R associations, we would expect participants to exhibit learning through motor responses alone, without any positive evidence from anticipatory gaze patterns. However, the presence of accurate gaze shifts before stimulus onset indicates that participants were predicting upcoming stimuli based on their perceptual processing of the sequence of stimuli, rather than simply creating associations between motor responses following stimulus presentation. Hence, the eye movement data from the *preview window* from Study 2 led us to reject the hypothesis that the learning effects observed in manual responses of both studies arose from R-R or S-R associations. Rather, we argue that subjects detected regularities in the string by forming S-S associations, reflecting learning based on the sequence of perceived stimuli. Indeed, if the learning were motor-based, we should not expect such results in anticipatory gaze shifts, as these are indicative of perceptual learning rather than motor learning. Thus, our findings strongly support the conclusion that the observed learning effects are driven by perceptual S-S associations.

As noted by [23], a potential concern is whether perceptual learning can be completely isolated from motor responses, particularly if oculomotor programming is considered a form of motor response. However, as pointed out in [23], research in the attention and oculomotor literature suggests that perceptual-based learning is more closely associated with shifts in visuospatial attention than with oculomotor programming. Specifically, eye movements are typically preceded by shifts in attention, indicating that attention is directed towards a stimulus before any eye movement occurs [23, 52–57]. Moreover, the mechanisms for directing attention and those for eye movements are distinct and operate independently, meaning that attention can be directed towards a stimulus without involving a motor response. This evidence supports the idea that perceptual-based learning can occur through shifts in attention alone, independent of motor responses [53, 55, 58–61]. Given these considerations, it is fair enough to conclude that perceptual-based learning does not rely on oculomotor programming. Instead, the automatic orienting of visuospatial attention, which can occur without eye movements, sufficiently accounts for the perceptual learning observed in our study. Thus, the anticipatory gaze shifts we measured are likely a reflection of perceptual processing rather than motor-based responses, reinforcing the conclusion that the learning effects are indicative of stimulus-stimulus (S-S) associations rather than motor or oculomotor factors.

This hypothesis is further supported by recent studies investigating and confronting implicit sequence learning (ISL) in the auditory and tactile sensory domains [45]. These studies found domain-specific differences, reinforcing the notion that the learning observed was perceptual in nature and related to specific stimuli rather than being purely motor-based. If the learning were entirely motor-based, we would not expect to detect variations in learning across different sensory modalities (visual, tactile, and auditory), especially since participants were required to press the same keys (the *z* and *m* keys on the keyboard) in response to stimuli of different types. Importantly, in both the tactile and auditory experiments, no anticipatory gaze shifts were observed, as participants were instructed to fixate on a central asterisk while auditory or tactile stimuli were presented. This design further supports the hypothesis that perceptual-based learning, rather than motor responses, accounts for the observed implicit statistical learning effects.

The question concerning the sources of the alternation advantage has never been properly addressed in previous studies, which have long considered this bias a guessing strategy of participants (among others, [2, 3, 62]. The only work dealing with the issue was [11], showing that the alternation advantage originates from perceptual rather than visuomotor processing, in that it follows the location of the previous stimulus and not that of the previous saccade, while not excluding a possible connection with IOR [16, 20, 21]. In this respect, the present work makes an important contribution to the topic: the investigation of this effect in two different time windows (before and after stimulus onset) and through different behavioural measures (manual RTs and saccades) has provided in fact novel insights on the nature of its components and on how it interacts with the learning process. Indeed, unlike [11], our stimulus sequence was not randomized but allowed the learning of underlying grammatical regularities. As discussed above, a bias toward alternation was found in manual responses in Study 1 with an advantage for the trigram 01**0** over 01**1**. Crucially, such effect was attested also in the oculomotor component of the task in Study 2, where we hypothesized that it also interacted significantly with the application of the First Law in both grammars. This evidence suggests that the alternation advantage is not confined to an alternation motor effect associated with manual responses as it has been attested for the first time also in the oculomotor predictive behaviour. Indeed, the alternation advantage was found in the preview window, which *precedes* the stimulus onset: participants directed their gaze toward the side of the screen where they expected the square to appear. On these grounds, we propose that shifts in visuospatial attention–occurring before and independently of oculomotor programming (see [23] for detailed discussion, and [63]’s *Visual Attention Model*), played a significant role in the occurrence of the alternation advantage observed in Study 2. Specifically, we argue that the spatial positioning of the stimulus is a key perceptual factor driving this effect. In other words, while the alternation advantage is triggered by the previous location of the stimulus (as suggested by [11]), it is closely related to the spatial properties of the stimuli, which are presented in opposing positions. This likely involves a mechanism that encourages broader exploration of the context, triggering shifts in attention even before the stimulus appears. This analysis strongly supports the central role of attentional mechanisms in driving the alternation advantage. Again, this hypothesis is further corroborated by [45]. While ISL was attested in both the auditory and tactile modalities, the alternation advantage was found in the tactile domain but not in the auditory one. Crucially, the major difference between the two studies lies in the fact that auditory stimuli differed solely on perceptual features (i.e., their Hz frequencies were simultaneously presented to both ears), while tactile stimuli were distinguished only based on *spatial* characteristics (i.e., vibrations of equal intensity and frequency were transmitted either to the left or the right thumb). This supports the primary role of the spatial dimension of stimuli in determining the alternation advantage. Moreover, it provides further evidence of the link between such effect and (visuo)spatial attention, and the independence of the latter from oculomotor programming. Indeed, the tactile study did not require the execution of any saccadic eye movement; rather, subjects were asked to provide manual responses while looking at a fixation cross appearing in the centre of the computer screen, simultaneously with the transmission of the tactile stimulus.

Another interesting aspect we tackled in the present work was the relationship between the alternation advantage and the statistical properties of stimuli in the strings. We wanted to verify the potential presence of the alternation advantage both in conditions in which there was a greater chance of having alternating (Skip blocks) and repeating stimuli (Fib blocks). Interestingly, we noted that performance was better overall on 01**0** than 01**1**, in both grammars. This manifested both in terms of RTs (Study 1) and rates of correct anticipations (Study 2). In other words, we found straightforward evidence of the presence of the alternation advantage, which manifested itself early on and persisted throughout the task, regardless of the distributional properties or the transitional probabilities of the two trigrams in Fib and Skip. Crucially, despite this, we noticed an inverse performance pattern on 01**0** and 01**1** in the two grammars. Specifically, RTs on 01**1** dropped significantly in Fib blocks, while conversely, those on 01**0** dropped significantly in Skip (Study 1). Moreover, we found that accuracy rates on anticipations significantly increased on 01**1** in Fib blocks, while significantly decreasing on 01**0** (Study 2), and, in the last Skip block, the trend reversed (although not significantly). This result might be the manifestation of the interference between the alternation advantage and statistical learning: although the alternation advantage persisted throughout the task, it might have been modulated by a statistical learning effect. Indeed, both the tracking of distributional patterns and the effect of transitional probabilities may have caused this result (see Section 2 and Section 2.1.3). However, other–more intriguing–explanations are also possible, based on the capacity of the human parser to build abstract hierarchical representations. [35, 45] propose a parsing strategy according to which the parser builds multiple layers of representation by recursively building deterministic transitions among progressively larger chunks. This hypothesis implies that low-level deterministic transitional regularities are used to form small chunks, which are then embedded into larger chunks, and in turn, these larger chunks are used to form even larger embedded chunks, involving the very same transitional probabilities. Given this kind of hierarchical computations, points that were originally not deterministically predictable on the string become progressively predictable, emerging as subparts of higher-level deterministic transitions. In [38] the authors explore a different hypothesis about the role played by structural reconstruction, based on the possibility that the human parser applies a wired-in mapping principle ensuring bootstrapping from linear order to hierarchical representations. The basic insight is that if x precedes y, then x must be contained in y. The formal application of this principle ensures that the deterministic chunk 0**1** be categorized as 1 at the higher level, whereas the 1s following 01 are categorized as 0s. It is easy to show that this strategy of *categorial labelling* produces precisely the same results, in Fib, as the structural strategy proposed in [35]. However, a possible advantage of the bootstrapping principle is that structural reconstruction might apply here as a sort of hypothetical reasoning: if 01 were a 1 at generation n-1, and this 1 were a 0 at generation n-2, then this 0 would be necessarily followed by a 1 at n-2, generating a 0 at n-1 and a 1 at the original n-level. In other words, hypothetical reasoning would predict a 1 after 01 in Fib, deriving the progressive decreasing of RTs in 01**1** as opposed to 01**0**, in line with our experimental findings for Fib. Conversely, in Skip, hypothetical reasoning makes a prediction that is discarded by the result of statistical analysis on the string (01 is more frequently followed by a 0 than by a 1).

Despite the important insights provided by our study and the findings discussed, there are also some limitations that warrant consideration. The first concerns the order of presentation of the two grammars in our studies and the potential priming effects this might have caused. Regarding this point, it could be argued that the order of presentation may have influenced our results. However, in [38], a reverse block order (Skip followed by Fib) was used, and the results were consistent with those found in this study. This supports the idea that the effects observed in the present study are more likely attributable to implicit statistical learning rather than potential priming effects. Based on these considerations, we believe that the order of grammar presentation does not significantly impact the results. Nonetheless, as suggested by a reviewer, future studies could further disentangle implicit statistical learning from possible priming effects by balancing block order between subjects. For instance, odd-numbered subjects could begin with three blocks of Fib grammar, while even-numbered subjects could start with three blocks of Skip grammar. Alternatively, separate groups exposed exclusively to either Fib or Skip blocks could provide a valuable approach to exclude potential priming effects. Another important point worth considering pertains to the dissociation between conscious and unconscious processes when assessing implicit learning. An anonymous reviewer raised the concern that verbal reports may be insufficient for evaluating implicit learning, as participants might struggle to verbalize their observations due to low confidence or unfamiliarity with the paradigm. To address this limitation, future studies could incorporate confidence ratings when noticing specific patterns [64], such as square sequences. This approach could provide a subjective yet more precise measure of awareness, helping to better distinguish between conscious and unconscious learning processes. In addition to these points, it goes without saying that further research–including a broader empirical base—is required to precisely assess the role of the different computational strategies subjects exploit in processing generations of symbols in Fib and Skip. We are confident, however, that the present study enlightens in some detail an important feature that these strategies seem to be endowed with: instead of being in competition with each other, they crucially interact with each other, some of them progressively weakening (such as the alternation bias), and some of them progressively gaining impetus (such as statistically-based (hierarchical) learning). This dynamic process is apparently governed by a unique higher-level strategy: optimizing computation to increase the prediction power of the parser.
